# The Giant *Cafeteria roenbergensis* Virus That Infects a Widespread Marine Phagocytic Protist Is a New Member of the Fourth Domain of Life

**DOI:** 10.1371/journal.pone.0018935

**Published:** 2011-04-29

**Authors:** Philippe Colson, Gregory Gimenez, Mickaël Boyer, Ghislain Fournous, Didier Raoult

**Affiliations:** 1 Unité de Recherche sur les Maladies Infectieuses et Tropicales Emergentes (URMITE), Centre National de la Recherche Scientifique (CNRS) Unité Mixte de Recherche (UMR) 6236 - Institut de Recherche pour le Développement (IRD) 3R198, Facultés de Médecine et de Pharmacie, Université de la Méditerranée, Marseille, France; 2 Pôle des Maladies Infectieuses et Tropicales Clinique et Biologique, Fédération de Bactériologie-Hygiène-Virologie, Centre Hospitalo-Universitaire Timone, Marseille, France; University of Kansas Medical Center, United States of America

## Abstract

**Background:**

A recent work has provided strong arguments in favor of a fourth domain of Life composed of nucleo-cytoplasmic large DNA viruses (NCLDVs). This hypothesis was supported by phylogenetic and phyletic analyses based on a common set of proteins conserved in *Eukarya*, *Archaea*, *Bacteria*, and viruses, and implicated in the functions of information storage and processing. Recently, the genome of a new NCLDV, *Cafeteria roenbergensis virus* (CroV), was released. The present work aimed to determine if CroV supports the fourth domain of Life hypothesis.

**Methods:**

A consensus phylogenetic tree of NCLDVs including CroV was generated from a concatenated alignment of four universal proteins of NCLDVs. Some features of the gene complement of CroV and its distribution along the genome were further analyzed. Phylogenetic and phyletic analyses were performed using the previously identified common set of informational genes present in *Eukarya*, *Archaea*, *Bacteria*, and NCLDVs, including CroV.

**Findings:**

Phylogenetic reconstructions indicated that CroV is clearly related to the *Mimiviridae* family. The comparison between the gene repertoires of CroV and Mimivirus showed similarities regarding the gene contents and genome organization. In addition, the phyletic clustering based on the comparison of informational gene repertoire between *Eukarya*, *Archaea*, *Bacteria*, and NCLDVs unambiguously classified CroV with other NCLDVs and clearly included it in a fourth domain of Life. Taken together, these data suggest that *Mimiviridae*, including CroV, may have inherited a common gene content probably acquired from a common *Mimiviridae* ancestor.

**Conclusions:**

This further analysis of the gene repertoire of CroV consolidated the fourth domain of Life hypothesis and contributed to outline a functional pan-genome for giant viruses infecting phagocytic protistan grazers.

## Introduction

In 2003, the discovery of *Acanthamoeba polyphaga* Mimivirus, which has the largest viral genome (1,18 kilobases (kb)) ever reported [1], [2], gave a boost to knowledge and understanding in terms of the definition and origin of viruses [3], [4]. Mimivirus was revealed as a new member of the nucleo-cytoplasmic large DNA viruses (NCLDVs) superfamily, a monophyletic group of viruses composed of the *Poxviridae*, *Phycodnaviridae*, *Irido-/Asco-viridae*, and *Asfarviridae* families [2], [5], [6]; additionally, Mimivirus was the first member of the new *Mimiviridae* family [2]. Between 2008 and 2009, Mamavirus, a very close relative of Mimivirus associated to the first ‘virophage’ Sputnik that infects these two giant viruses, and Marseillevirus, a new giant virus, were isolated from *Acanthamoeba* spp. and were classified within the NCLDV lineage [7], [8]. NCLDVs infect various eukaryotic hosts including vertebrates, insects, algae, or protists [9]–[11]. Recently, Yutin et al. identified a set of 47 conserved genes among NCLDVs (NCLDV core genes) from the construction of clusters of orthologous NCLDV genes (NCVOGs) [6].

The isolation of Mimivirus and the analysis of its genome have contributed to the emergence or revival of groundbreaking paradigms that put forward giant viruses as possible major ancestors in the early steps of Life evolution [2], [4], [12], [13]. Thus, Mimivirus has been suspected to constitute a fourth domain of Life, apart from bacteria, archaea and eukaryotes, based on the phylogeny of some of the *Mimiviridae* genes that are parts of a group of seven genes encoding universal proteins [2]. This assumption has been vigorously debated though discussion of the appropriateness of genes used and the interpretation of phylogeny reconstructions [2], [13]–[17].

In a recent paper, Boyer et al. provided strong arguments in favor of the existence of this fourth domain of Life. This hypothesis was supported, on the one hand, by phylogenetic analysis of eight proteins implicated in the functions of information storage and processing and highly conserved among eukaryotes, archaea, bacteria, and viruses, and on the other hand, by phyletic studies of their repertoire of informational genes [12]. This work was successfully achieved with the availability of new genomes of giant viruses, including Marseillevirus that has been recently isolated from amoeba and that could represent a new NCLDV family [8], [18]. The genome of a new giant virus, *Cafeteria roenbergensis* virus (CroV), was recently released [19]. This virus infects the phagocytic protist *Cafeteria roenbergensis*, a widespread marine heterotrophe flagellate that belongs to the *Chromalveolata* phylum and that is therefore phylogenetically very distant from the amoebal host of Mimivirus and Marseillevirus, *Acanthamoeba* spp; nonetheless, those phagocytic protists graze on bacteria and viruses [1], [8], [18], [19], [20]. The CroV genome has an estimated size of 730 kb, contains 544 putative ORFs, and is therefore the second largest among currently available viruses. The availability of this new giant viral genome represents a remarkable opportunity to investigate the existence of a pan-genome of giant viruses of protists, including CroV, which has been recovered from a different geographical area and a different environmental water sample than Mimivirus and Marseillevirus. In the present work, we sought to re-analyse the proteome of CroV, paying very close attention to its comparison with that of Mimivirus, and to determine if it supports the fourth domain of Life hypothesis.

## Results

### Consensus phylogenetic tree of the NCLDVs

**Figure 1:**
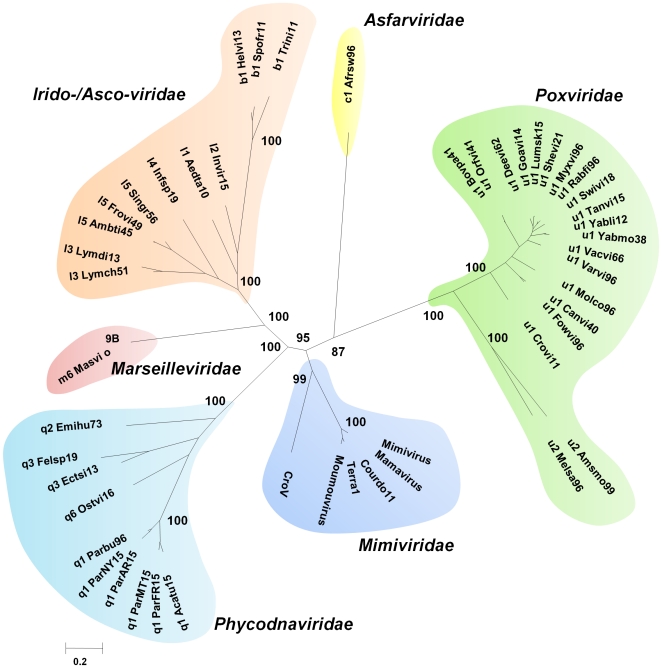
Consensus phylogenetic tree of the NCLDVs. Bayesian phylogenetic tree was constructed from a cured concatenated alignment of 4 universal NCVOGs (496 conserved positions), including CroV corresponding proteins: primase-helicase (NCVOG0023), DNA polymerase (NCVOG0038), packaging ATPase (NCVOG0249), and A2L-like transcription factor (NCVOG0262). Bayesian posterior probabilities are mentioned near branches as a percentage and are used as confidence values of tree branches. Only probabilities at major nodes are shown. Scale bar represents the number of estimated changes per position for a unit of branch length. *Abbreviated names for NCLDVs*: b1_Helvi, Heliothis virescens ascovirus 3e; b1_Spofr, Spodoptera frugiperda ascovirus 1a; b1_Trini, Trichoplusia ni ascovirus 2c; c1_Afrsw, African swine fever virus; l1_Aedta, Aedes taeniorhynchus iridescent virus (Invertebrate iridescent virus 3); l2_Invir, Invertebrate iridescent virus 6; l3_Lymch, Lymphocystis disease virus - isolate China; l3_Lymdi, Lymphocystis disease virus 1; l4_Infsp, Infectious spleen and kidney necrosis virus; l5_Ambti, Ambystoma tigrinum virus; l5_Frovi, Frog virus 3; l5_Singr, Singapore grouper iridovirus; m6_Masvi, Marseille virus; q1_Acatu, Acanthocystis turfacea Chlorella virus 1; q1_ParAR, Paramecium bursaria Chlorella virus AR158; q1_Parbu, Paramecium bursaria Chlorella virus 1; q1_ParFR, Paramecium bursaria Chlorella virus FR483; q1_ParMT, Paramecium bursaria chlorella virus MT325; q1_ParNY, Paramecium bursaria Chlorella virus NY2A; q2_Emihu, Emiliania huxleyi virus 86; q3_Ectsi, Ectocarpus siliculosus virus 1; q3_Felsp, Feldmannia species virus; q6_Ostvi, Ostreococcus virus OsV5; u1_Bovpa, Bovine papular stomatitis virus; u1_Canvi, Canarypox virus; u1_Crovi, Crocodilepox virus; u1_Deevi, Deerpox virus W-848-83; u1_Fowvi, Fowlpox virus; u1_Goavi, Goatpox virus Pellor; u1_Lumsk, Lumpy skin disease virus NI-2490; u1_Molco, Molluscum contagiosum virus; u1_Myxvi, Myxoma virus; u1_Orfvi, Orf virus, complete genome; u1_Rabfi, Rabbit fibroma virus; u1_Shevi, Sheeppox virus 17077-99; u1_Swivi, Swinepox virus; u1_Tanvi, Tanapox virus; u1_Vacvi, Vaccinia virus; u1_Varvi, Variola virus (smallpox virus); u1_Yabli, Yaba-like disease virus; u1_Yabmo, Yaba monkey tumor virus; u2_Amsmo, Amsacta moorei entomopoxvirus; u2_Melsa, Melanoplus sanguinipes entomopoxvirus.

The phylogenetic tree of the NCLDVs based on a concatenated alignment of 4 universal NCVOGs (primase-helicase, DNA polymerase, packaging ATPase, and A2L-like transcription factor) indicated that CroV is related with the *Mimiviridae* family (Figure 1). Nonetheless, CroV is deeply positioned in the *Mimiviridae* branch, which contains a cluster formed by Mimivirus and all four close relative to Mimivirus isolated from *Acanthamoeba* spp. culture in our lab [18]. This phylogenetic reconstruction based on this very restricted but conserved set of genes suggests that two subfamilies may exist within the *Mimiviridae* family, one represented by Mimivirus and its very close relatives, and another represented by CroV.

### Comparison of gene content and architecture between CroV and *Mimiviridae* genomes

**Figure 2:**
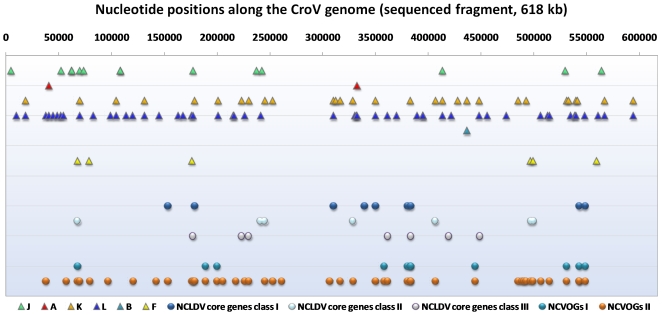
Genome map of the distribution of ORFs along the CroV genome (sequenced fragment, 618 kb). COG functional categories, NCLDV core genes (classes I–III), and NCVOGs.

Fischer et al. identified the presence of all 9 universal NCLDVs genes in the CroV genome [5], [19]. Recently, the latest update of the universal NCLDVs genes showed the existence of a set of 47 NCLDVs core genes [6], Among them, only 5 remained common to all NCLDVs, and they were also found in CroV (Table S1; Figure 2). By contrast, 14 CroV ORFs are lacking among those belonging to this set of 47 core genes, including notably four proteins implicated into DNA replication, recombination and repair, or nucleotide metabolism. Additionally, a RNA ligase and a dUTPase are present in CroV while absent in Mimivirus, and four additional proteins absent in Mimivirus (including an ATP-dependent DNA ligase and a thymidylate kinase) are also absent in CroV. Also, amongst the set of 47 NCLDVs core genes, eight out of the 18 that are absent in Marseillevirus are present in CroV. Regarding the 177 NCVOGs represented in two or more NCLDV families according to Yutin et al.'s study [6], 59 do not show the same pattern of presence/absence in CroV or Mimivirus (Table S2). In four cases (dUTPase, an adenine-specific DNA methyltransferase, an ubiquitin, and a RNA ligase), NCVOGs are found in CroV but not in Mimivirus; conversely, 55 NCVOGs are present in Mimivirus and absent in CroV. Thus, overall, substantial differences can be noted between the gene repertoires of both viral genomes regarding NCVOGs.

**Figure 3:**
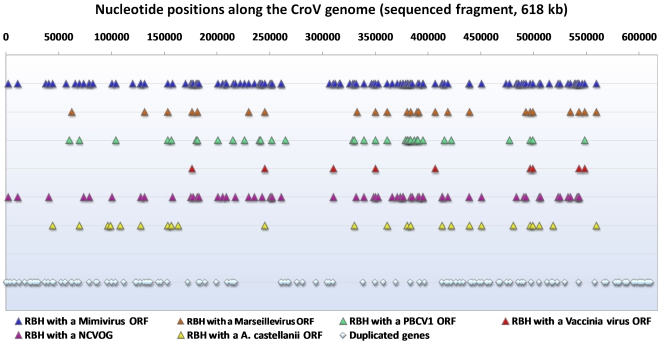
Genome map of the distribution of ORFs along the CroV genome (sequenced fragment, 618 kb). ORFs that form pairs of reciprocal best BLASTp hits with ORFs of Mimivirus, Marseillevirus, *Paramecium bursaria Chlorella* virus 1, Vaccinia virus, or *Acanthamoeba castellanii*, and duplicated genes. RBH, reciprocal best BLASTp hits.

**Figure 4:**
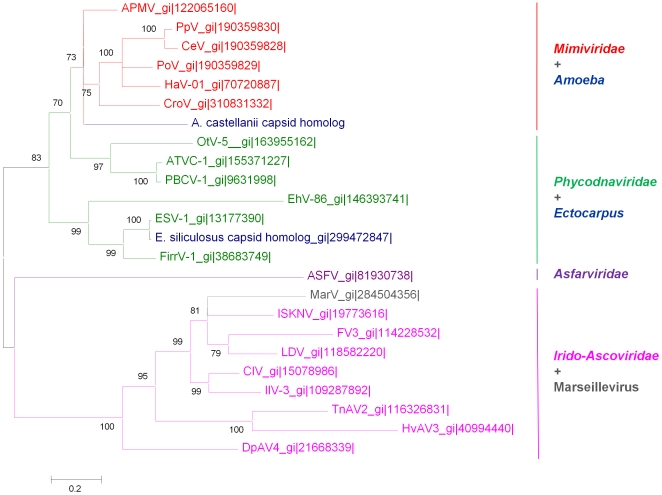
Phylogenetic tree of the NCLDV major capsid protein (MCP). The MCP phylogenetic tree was inferred with Bayesian approach from a cured alignment of 24 sequences (132 conserved positions) from the NCLDV families, from the *A. castellanii* capsid homolog fragment, and from the *Ectocarpus siliculosus* capsid homolog. Poxviruses capsid proteins were not included in the tree as their sequences are too divergent from those of other NCLDV families. Bayesian posterior probabilities are mentioned near branches as a percentage and are used as confidence values of tree branches. Scale bar represents the number of estimated changes per position for a unit of branch length. Abbreviations: APMV, *A. polyphaga* mimivirus; ASFV, African swine fever virus; ATCV-1, *Acanthocystis turfacea* chlorella virus 1; CeV, *C. ericina* virus 01; CIV, Chilo iridiscent virus; CroV, *C. roenbergensis* virus; DpAV4, *Diadromus pulchellus* ascovirus 4a; EhV-86, *E. huxleyi* virus 86; ESV-1, *Ectocarpus siliculosus* virus 1; FirrV-1, *Feldmannia irregularis* virus 1; FV3, Frog virus 3; HaV, *H. akashiwo* virus 01; HvAV3, *Heliothis virescens* ascovirus 3e; IIV-3, Invertebrate iridiscent virus 3; ISKNV, Infectious spleen and kidney necrosis virus; LDV, Lymphocystis disease virus; MarV, Marseillevirus; OtV-5, *Ostreococcus tauri* virus 5; PBCV-1, *P. bursarium* chlorella virus 1; PoV, *P. orientalis* virus 01; PpV, *P. pouchetti* virus 01; TnAV2, *Trichoplusia ni* ascovirus 2c. GI numbers are listed next to abbreviations of corresponding taxonomic name of each virus.

Proportions of ORFans (i.e. ORFs that lack homologs in the NCBI protein sequence database; e-value<1e-5) in the CroV and Mimivirus genomes are high and of the same order of magnitude, being 47.4% and 48.1%, respectively [21], [22] (Figure S1). Additionally, proportions of meta-ORFans, defined as ORFans that have homologs in environmental databases, are similar for the CroV and the Mimivirus genomes, being 8.8% and 6.9%, respectively [22]. Interestingly, 24 CroV proteins have a significant BLASTp hit (e-value<1e-5) with a putative ORF inferred from the genome of *Acanthamoeba castellanii* (Figure 3). Nineteen of these ORFs are attributed to NCVOGs, and in nine cases their predicted functions are related to DNA replication, recombination and repair, transcription and RNA processing, or nucleotide metabolism. Surprisingly, the NH2 terminal half of the major capsid protein (MCP) of CroV (first 240 amino acid residues of the 506 amino acids length protein) was found within the genome of *Acanthamoeba castellanii* (identity, 39%; expected value, 1e-44; Figure S2). Moreover, BLASTp searches for the CroV MCP against the NBCI non redundant protein sequence database showed a MCP homolog in *Ectocarpus siliculosus* proteome, in addition to those detected in NCLDVs. Noteworthy, phylogenetic reconstruction based on the NCLDVs capsid proteins showed that the *A. castellanii* capsid homolog fragment was clustered with the *Mimiviridae* capsid proteins, suggesting horizontal transfer of the MCP gene between this amoebal host and the *Mimiviridae* (Figure 4). We assumed that this transfer was ancient as the *A. castellanii* capsid homolog fragment showed a divergent sequence in comparison with those of the *Mimiviridae*. Besides, the MCP of *Ectocarpus siliculosus* Virus (EsV-1) was clustered with the capsid homolog fragment of *Ectocarpus siliculosus*, a brown alga and the host of EsV-1, but the two sequences showed a far lower divergence than for the previous case.

CroV ORFs that share a reciprocal best BLASTp hit with ORFs of a short panel of other NCLDVs or with NCVOGs tend to localize out of the ends of the viral genomes (Figure 3). Indeed, these ORFs are statistically significantly less frequent in the first and last 50,000 nucleotide-length fragments of the sequenced genome when involved in a pair of ORFs with Mimivirus (5.8% vs 22.7%, p = 0.00033), Marseillevirus (0% vs 5.2%, p = 0.015), *Paramecium bursaria Chlorella* virus (PBCV1) (0.0% vs 7.4%; p = 0.010), and the set of NCVOGs (3.5% vs 12.0%, p = 0.019). In contrast, duplicated genes are statistically significantly more frequent at the terminal parts of the CroV genome (20.1% vs 54.7%; p<1e-6) (Figure 3; Figure S3). Several syntenic organizations involving notably nine informational genes can be detected by genome comparison of CroV and Mimivirus (Table S3; Figures S4, S5, S6). They tend to locate outside the terminal parts of the genomes, and high scores of homology are observed for ORFs in synteny within the region of the genomes corresponding to nucleotides 150,000–500,000. Taken together, these results indicate similarities regarding the maps of the CroV and the *Mimiviridae* genomes. Besides, codon usage is similar between CroV and Mimivirus (Figure S7).

### Phylogenetic and phyletic analysis of informational genes

**Figure 5:**
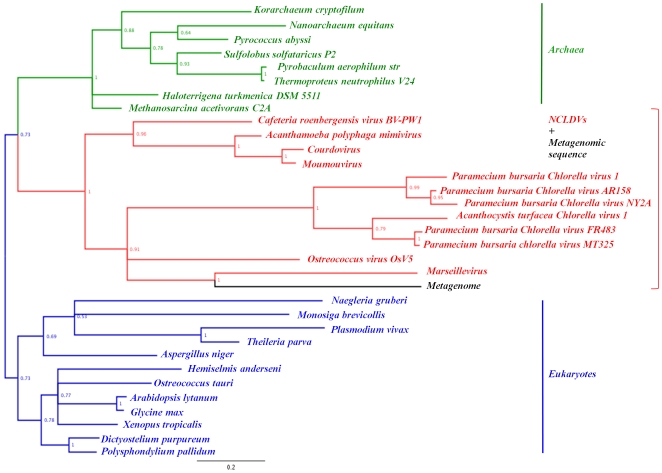
TFIIB (transcription factor II B) phylogenetic tree (82 sequences, 97 positions) constructed using a Bayesian approach. Bayesian posterior probabilities are mentioned near branches and are used as confidence values of tree branches. A color code was used to represent taxonomic groups, *Archaea* in green, *Eukarya* in blue, NCLDVs in red, and environmental sequences in black. For details on evolutionary models and phylogenetic methods, see Materials and Methods.

Phylogenetic reconstructions based on the ribonucleotide reductase (RNR; Figure S8), the DNA polymerase family B (DNApol; Figure S9), the proliferating cell nuclear antigen (PCNA; Figure S10), the Flap endonuclease (FEN; Figure S11), and the transcription factor II B (TFIIB; Figure 5) show a clustering of the CroV proteins with their respective Mimivirus homologs, and with that of other *Mimiviridae* for TFIIB. In contrast, phylogenies for the DNA-dependant RNA polymerase II (RNAP II; Figure S12) show that CroV RNAP II is more related with the *Irido-/Asco-viridae* clade. Phylogeny reconstructions for the thymidylate synthase (ThyA) and the topoisomerase II A (TopoIIA) are not shown because they were not sufficiently resolved to provide strong support regarding the CroV phylogenetic position; nevertheless, their phylogenetic trees indicate CroV clustering with Marseillevirus and metagenomic sequences, respectively. Overall, phylogenetic reconstruction based on each of the eight proteins from this set are highly similar to those previously published by Boyer et al. [12]. Taken together, our results indicate that CroV is likely a *bona fide* new member of the *Mimiviridae* family within the NCLDV superfamily. Such a relationship was not observed in all phylogenetic trees, possibly because genomes of CroV and other giant viruses associated with protists were greatly affected by horizontal gene transfers (HGTs) and non-orthologous gene displacements [19], [23], [24].

**Figure 6:**
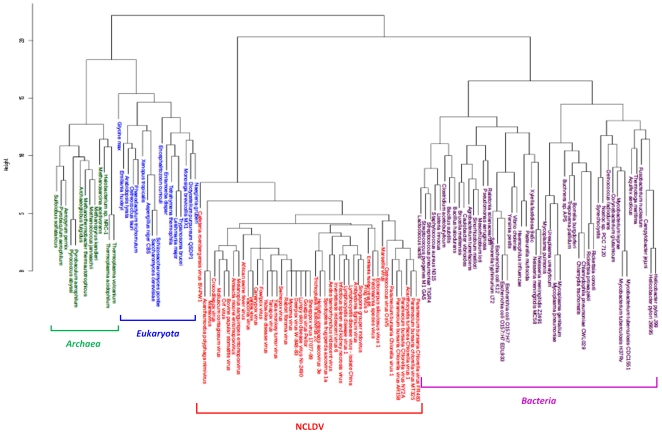
Hierarchical clustering of *Eukarya*, *Bacteria*, *Archaea* and NCLDVs by phyletic patterns. The phyletic patterns of the putative orthologous sets of informational genes indicating the presence/absence of the respective gene in each cellular organism and virus were used for the construction of the dendogram tree. For details, see Materials and Methods. A color code was used to represent taxonomic groups, *Bacteria* in purple, *Archaea* in green, *Eukarya* in blue, and NCLDVs in red.

**Figure 7:**
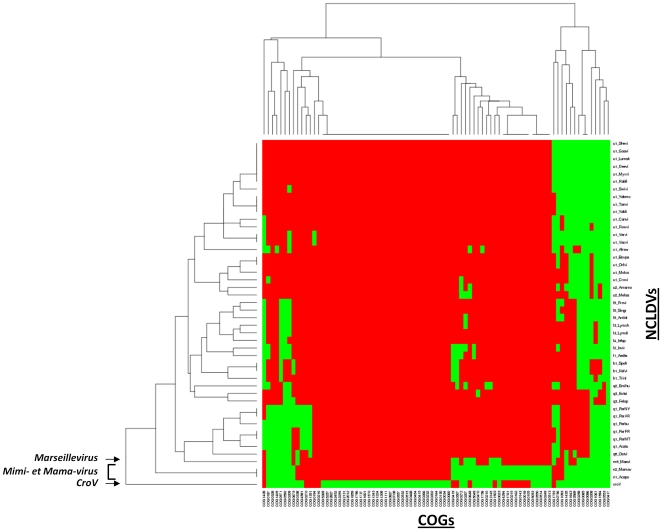
Heatmap built from the hierarchical clustering on the Euclidean distance computed from a presence/absence matrix based on COGs at least present in one NCLDV genome. Green, presence; red, absence. *Abbreviated names for NCLDVs*: b1_Helvi, Heliothis virescens ascovirus 3e; b1_Spofr, Spodoptera frugiperda ascovirus 1a; b1_Trini, Trichoplusia ni ascovirus 2c; c1_Afrsw, African swine fever virus; l1_Aedta, Aedes taeniorhynchus iridescent virus (Invertebrate iridescent virus 3); l2_Invir, Invertebrate iridescent virus 6; l3_Lymch, Lymphocystis disease virus - isolate China; l3_Lymdi, Lymphocystis disease virus 1; l4_Infsp, Infectious spleen and kidney necrosis virus; l5_Ambti, Ambystoma tigrinum virus; l5_Frovi, Frog virus 3; l5_Singr, Singapore grouper iridovirus; m6_Masvi, Marseille virus; n1_Acapo, Acanthamoeba polyphaga mimivirus; n2_Mamav, Mamavirus; q1_Acatu, Acanthocystis turfacea Chlorella virus 1; q1_ParAR, Paramecium bursaria Chlorella virus AR158; q1_Parbu, Paramecium bursaria Chlorella virus 1; q1_ParFR, Paramecium bursaria Chlorella virus FR483; q1_ParMT, Paramecium bursaria chlorella virus MT325; q1_ParNY, Paramecium bursaria Chlorella virus NY2A; q2_Emihu, Emiliania huxleyi virus 86; q3_Ectsi, Ectocarpus siliculosus virus 1; q3_Felsp, Feldmannia species virus; q6_Ostvi, Ostreococcus virus OsV5; u1_Bovpa, Bovine papular stomatitis virus; u1_Canvi, Canarypox virus; u1_Crovi, Crocodilepox virus; u1_Deevi, Deerpox virus W-848-83; u1_Fowvi, Fowlpox virus; u1_Goavi, Goatpox virus Pellor; u1_Lumsk, Lumpy skin disease virus NI-2490; u1_Molco, Molluscum contagiosum virus; u1_Myxvi, Myxoma virus; u1_Orfvi, Orf virus, complete genome; u1_Rabfi, Rabbit fibroma virus; u1_Shevi, Sheeppox virus 17077-99; u1_Swivi, Swinepox virus; u1_Tanvi, Tanapox virus; u1_Vacvi, Vaccinia virus; u1_Varvi, Variola virus (smallpox virus); u1_Yabli, Yaba-like disease virus; u1_Yabmo, Yaba monkey tumor virus; u2_Amsmo, Amsacta moorei entomopoxvirus; u2_Melsa, Melanoplus sanguinipes entomopoxvirus.

Regarding the phyletic study comparing informational gene repertoires between bacteria, archaea, eukaryotes and NCLDVs, the topology of the resulting dendrogram unambiguously shows a clade including CroV and the other NCLDVs (Figure 6). This clade is clearly distinct of three other ones that include respectively organisms of the three canonical domains of Life. Moreover, by focusing particularly on the position of CroV in this cladogram, we noticed that CroV branched deeply inside the NCLDV clade, in contrast to the phylogenetic tree of the NCLDVs universal genes, which puts CroV together with the *Mimiviridae*. Further analysis evidenced that 83 informational proteins corresponding to COGs at least present in one NCLDV genome account for the distance of CroV from the other NCLDVs (Figure 7; Table S4). Actually, among these 83 proteins, we found that 16, 1, 33, and 12 CroV proteins showed bacterial, archaeal, eukaryotic, and viral homologs, respectively (Figure S1). Additionally, 47 of these 83 proteins are differently represented in CroV and Mimivirus genomes. Amongst these 47 proteins, we could underline that an isoleucyl-tRNA synthetase, a ribosome-associated chaperone zuotin, two histone acetyltransferases and a deoxyribodipyrimidine photolyase were specifically found in CroV. Taken together, previous findings indicate that the CroV and Mimivirus gene repertoires are related. Besides, their sympatric lifestyle into phagocytic protistan grazers and their specific ecolological niche probably led to shape their respective genome with genes acquired from distinct sources.

## Discussion

Our present analysis identified similar features for CroV and Mimivirus genomes, with a fully conserved albeit very limited core gene set (corresponding to the nine NCLDVs core genes or the five NCVOGs shared by all NCLDVs [6]), and substantial numbers of duplicated genes, genes putatively exchanged by HGTs, and ORFans and meta-ORFans; in addition, the gene content and its organization along the CroV genome is similar to what has been observed for Mimivirus [2], [24]–[26].

Interestingly, CroV ORFs that share a reciprocal best BLASTp hit with ORFs of other NCLDVs (Mimivirus, Marseillevirus, and even a phycodnavirus) are statistically significantly less frequent at both 50 kb-ends of the sequenced genome of CroV, whereas duplicated genes are statistically significantly more frequent in these regions toward the terminal parts of the genome. Moreover, these observations should take into account that among the nearly 730 kb of the CroV genome, only the 618-kb central part has been sequenced, with the terminal parts of the genome being described as highly repetitive regions [19]. Such an uneven distribution has been underscored previously in the Mimivirus genome. Thus, lineage-specific gene extension has been rather described within the first and last 200 kb of Mimivirus [26]. Moreo*v*er, HGTs between bacteria and Mimivirus tend to be localized at the ends of the Mimivirus genome [24]. Concerning other large eukaryotic DNA viruses, Schakelton and Holmes have hypothesized that core viral genes inherited vertically from an ancient viral ancestor might be located in the central part of the genome, while genes involved in (HGTs with cellular host species or other viruses might localize at the ends of the genome [27]. Additionally, an analysis of 20 poxvirus genomes found that orthologs were centrally located, whereas genes unique to a given species were located at the terminal parts of the genome [28]. Also, homologous recombination in poxviruses was shown to occur at the ends of the viral genome in most cases [29]. Taken together, the previous findings prompt to determine if similar features will be also observed in the genomes of giant viruses that will be available in the future. An unexpected result was the identification of an *A. castellanii* sequence coding a putative protein homologous to the N-terminal part of the CroV and *Mimiviridae* MCP. This finding suggests gene transfers between CroV or related giant viruses and their host. Notably, previous studies suggested that the genome of *Ectocarpus siliculosus* virus-I (EsV-I) may be integrated into the DNA of its host, a marine filamentous brown alga [30]. Because the genome of *Cafeteria roenbergensis* is unavailable, no genome comparison could be performed to detect HGTs between CroV and its host. However, sequence similarity detected between CroV MCP and an *A. castellanii* sequence suggests that phagocytic protists like amoebae may have represented the ancestral host of CroV, which may have subsequently specialized to infect flagellate protists.

In phylogenetic analysis performed on several conserved genes or concatenation of universal NCLDV genes in Fischer et al.'s study [19] or in the present work, CroV was in most cases found in the same clade as the *Mimiviridae*. This is true for instance when using the MCP or the DNA polymerase family B. In some other cases, the phylogenetic link between CroV and *Mimiviridae* is less robust. Some CroV genes are clustered with other NCLDVs or even homologs from other organisms outside the NCLDVs lineage. For instance, in the present study, phylogenetic analysis of RNA polymerase II showed that CroV is clustered with irido-/asco-viruses, while in Fischer et al.'s study, phylogenetic analysis of KDO 8-P phosphatases or of arabinose-5-phosphate isomerases showed that CroV proteins tend to be clustered with bacterial homologs. Such cases likely indicate the complex and mosaic gene content of giant viruses infecting protists; classically, trees topology might be blurred by HGT [31]. Besides, substantial differences between the genomes of CroV and Mimivirus have been shown by Fischer et al. and by our study, which may question if CroV is a new bona fide *Mimiviridae*. Two major findings from the present study can be underscored regarding this issue. First, the consensus phylogenetic tree of the NCLDVs based on a concatenated alignment of four universal NCVOGs showed that the CroV branch emerges before the *Mimiviridae* cluster. This suggests that CroV on the one hand and Mimivirus and its close relatives on the other hand form two sub-families within the *Mimiviridae*. Nevertheless, this finding relies on about only 1% of the gene content of CroV. Phylogenetic analysis indicated that CroV-Mimivirus phylogenetic separation probably occurred very anciently. Hence, the genomes of CroV and Mimivirus could have undergone specific genetic rearrangements. In addition, HGTs could have occurred from distinct sources as CroV and Mimivirus infect a marine flagellate and a soft water amoeba, respectively, which are distantly related and might host different sympatric bacterial and viral intracellular communities. Second, the phyletic analysis based on informational COGs also showed substantial differences between the gene contents of CroV and Mimi- or Mamavirus. Notwithstanding, four domains of Life were clearly delineated based on this dataset, and CroV was located unambiguously within the same domain as other NCLDVs.

Thus, a major finding of the present work is that the gene content of CroV unambiguously allows its classification as a new member of the fourth domain of Life, along with other NCLDVs, which confirms and extends the results recently published by Boyer et al. [12]. Indeed, the phylogenetic and phyletic analysis of proteins involved in the storage and processing of information, including those of CroV, confirms the existence of a common set of ancestral genes among NCLDVs. Therefore, the present work supports the monophyletic origin for this group of viruses, and the existence of a core genome for NCLDVs, as previously described [5], [6], [32]. Taken together, the conservation in NCLDVs of a set of genes encoding informational proteins, which constitutes a relatively stable backbone as observed in the gene contents of *Bacteria*, *Archaea*, and *Eukarya*, and indications of common ancestors harboring these genes justify defining a fourth domain of Life. The validation of this latter hypothesis *a posteriori* by taking into account CroV proteins confers it with increased robustness.

In conclusion, analysis of the gene repertoire of CroV consolidated the fourth domain of Life hypothesis and contributed to outline a functional pan-genome for NCLDVs replicating in phagocytic protistan grazers. Nonetheless, the sympatric lifestyle of giant viruses infecting phagocytic protists might lead them to have complex and chimeric genomes, with genes of various origins, acquired from bacteria, archaea, eukaryotes, and viruses. Finally, taken together, previous data provide additional evidence that viruses, at least NCLDVs, are not minor elements of the biosphere.

## Materials and Methods

### Consensus phylogenetic tree of the NCLDVs

The consensus phylogenetic tree of the NCLDVs was obtained from a tree constructed from a concatenated alignment of four universal NCVOGs [6]: primase-helicase (NCVOG0023), DNA polymerase (NCVOG0038), packaging ATPase (NCVOG0249), and A2L-like transcription factor (NCVOG0262). Multiple sequence alignments and phylogenetic reconstruction were performed as previously described [12]. Briefly, multiple sequence alignments were generated using T-Coffee [33] and conserved blocks were selected using Gblocks [34]. Thereafter, phylogeny was based on the Bayesian inference (BI) approach using MrBayes [35]. The WAG matrix was used, and model parameters (gamma shape and proportion invariant) were allowed to vary through the Markov Chain Monte Carlo Chain (MCMC). Four MCMC chains were run for 500,000 generations and sampled every 100th generation. The first 100,000 trees were discarded, and the sumt command of MrBayes was used to compute clade posterior probabilities. Trees were displayed using MEGA 4 [36].

### Analysis of specific features of the gene complement of CroV

The gene content of CroV and that of other NCLDVs were compared using the best reciprocal BLASTp hits strategy, allowing the detection of pairs of ORFs that are reciprocal best hits for each other [37], [38]. Expected value (e-value) cutoff for this analysis was 1e-5. BLASTp was also performed with a e-value of 1e-5 against the NCBI environmental database (env_nr). Additionally, the CroV proteome was compared using BLASTp with that of *Acanthamoeba castellani* inferred by GeneMark [39] using nucleotide sequences available from ftp://ftp.hgsc.bcm.tmc.edu/pub/data/AcastellaniNeff/ (Baylor College of Medicine, human genome sequencing center). Relative synonymous codon usage (RSCU) of each CroV gene was calculated using the CAIcal web-server (http://genomes.urv.cat/CAIcal) [40]. Genomic maps corresponding to the distribution of the CroV gene content along the genome were plotted using Microsoft Excel software. Positions within the genome were determined for CroV ORFs involved in pairs that are reciprocal best BLASTp hits for each other using the proteomes of Mimivirus, Marseillevirus, *Paramecium bursaria Chlorella* virus 1 (PBCV-1), a phycodnavirus, and Vaccinia virus, a poxvirus. Also, NCLDV core genes, ORFs corresponding to COGs and NCVOGs, duplicated genes, ORFans and meta-ORFans, and ORFs classified on the basis of the taxonomy of their best BLASTp hits in the NCBI non-redundant protein sequence database were plotted according to their position on the genome. Duplicated genes were tentatively identified by BLASTp searches for the CroV proteome against itself and considering significant hits with evalue<1e-10; the annotations of these genes were thereafter compared with that described by Fischer et al. as corresponding to duplicated genes [19]. The synteny of ORFs in the genomes of Crov and of *Mimiviridae* was determined using Microsoft Excel by sorting CroV's ORFs by their coordinates and calculating the relative positions of their best BLASTp hits in the other viral genome, or visually by building dot plots from pairs of viral proteins.

### Phylogenetic and phyletic analysis of informational genes

The present work was conducted accordingly to the same strategy and with the same sets of sequences, incremented with CroV proteins, as the recently published work of Boyer et al. [12]. Briefly, phylogenetic reconstructions were built using the proteomes of several selected organisms representing major phyla of the three domains of Life (*Eukarya*, *Archaea*, and *Bacteria*), and BLASTp searches were performed against the proteomes of viruses belonging to the NCLDV superfamily (*Asfarviridae*, *Asco-Iridoviridae*, *Phycodnaviridae*, *Poxviridae*, *Mimiviridae*, and Marseillevirus), the partial and draft proteomes of three new *Mimiviridae* [7], [18] and the proteome of CroV (GenBank accession number NC_014637.1). Indeed, the CroV proteome included the same set of eight proteins conserved among viruses from at least the *Mimiviridae* family among NCLDVs and at least two of the three domains of Life: thymidylate synthase (ThyA), ribonucleotide reductase (RNR), DNA polymerase family B (DNAP B), topoisomerase II A (TopoIIA), DNA-dependant RNA polymerase II (RNAP II), Flap endonuclease (FEN), processing factor Proliferating Cell Nuclear Antigen (PCNA), and Transcription factor II B (TFIIB). Homologs detected using BLASTp within the NCBI environmental non-redundant protein sequence database (env_nr) were also analyzed. Sequences were considered homologous if the best BLASTp hit (BBH) showed an alignment length >70 amino acid residues and a percent identity >20%. Multiple sequence alignments were generated using T-Coffee [33] or Muscle [41]; conserved blocks were identified with Gblocks [34] and alignments were manually curated. Phylogenetic reconstructions were thereafter built using BI, using the same tools and parameters as described previously [12] or to build the consensus phylogenetic tree of the NCLDVs.

Besides, comparison of informational gene repertoires was performed as previously described [12]. Thus, a phyletic pattern was used that indicates the presence or absence of genes from all species present in functional categories of COGs corresponding to information storage and processing (functional categories J, A, K, L, and B) or nucleotide transport and metabolism (functional category F) [42]. These sets of proteins were incremented with NCVOGs, proteins of 14 additional eukaryotic proteomes, and proteins of CroV corresponding to BLASTp hits with an e-value<1e-3 against the database of these COGs. The Euclidian distance matrix was computed from the 0/1 matrix obtained by assigning “1” if there is at least one ortholog in a genome or “0” if not. It allowed building a dendrogram tree generated from the hierarchical clustering using in house scripts in R language. Additionally, a presence/absence matrix based on COGs at least present in one NCLDV genome was generated using an in-house Perl script, then a heatmap was built from the hierarchical clustering on the Euclidean distance computed from this matrix by in-house R scripts.

## Supporting Information

Figure S1 **Genome map of the distribution along the CroV chromosome (sequenced fragment, 618 kb) of CroV ORFs assigned to COGs, and of ORFans and meta-ORFans.** Taxonomy for the best BLASTp hits against the NCBI non-redundant protein sequence database are indicated for all CroV ORFs assigned to COGs, and for those corresponding to COGs found discriminant among NCLDVs in the phyletic analysis.
(TIF)

Figure S2 **Alignment of amino acid sequences corresponding to the capsid protein of** ***Acanthamoeba polyphaga*** **Mimivirus (YP_142795.1; MIMI_R441), the major capsid protein of** ***Cafeteria roenbergensis*** **virus (YP_003969975.1; crov342), and** ***Acanthamoeba castellanii*** **.** Schematic of the alignment was obtained using Genedoc [http://www.nrbsc.org/gfx/genedoc/].
(TIF)

Figure S3 **Dot plot showing positions of duplicated genes (CroV ORFs with significant BLASTp hits against the CroV proteome) on the CroV genome.**
(TIF)

Figure S4 **Dot plot of scores for reciprocal BLASTp hits (e-value<1e-4) between Mimivirus and CroV ORFs using bl2seq (** www.ncbi.nlm.nih.gov/blast/bl2seq/wblast2.cg **).** Pink hachured lines delineate the region of the CroV genome of bacterial origin.
(TIF)

Figure S5 **Dot plot of Mimivirus and CroV ORFs sharing reciprocal BLASTp hits (e-value<1e-100), as determined using bl2seq, and belonging to groups of at least two successive ORFs in synteny.** Pink hachured lines delineate the region of the CroV genome of bacterial origin.
(TIF)

Figure S6 **Nucleotide dot plot for windows of 30 nucleotides for regions corresponding to Mimivirus ORFs 453 to 440 and CroV ORFs 160 to 178.**
(TIF)

Figure S7 **Relative synonymous codon usage (RSCU) for CroV and Mimivirus ORFs.**
(TIF)

Figure S8 **Phylogenetic trees constructed using a Bayesian approach are shown here and in Figures S9, S10, S11, and S12.** This is an RNR (ribonucleotide reductase) phylogenetic tree (32 sequences, 214 positions). Bayesian posterior probabilities are mentioned near branches and are used as confidence values of tree branches. A color code was used to represent taxonomic groups, Bacteria in purple, Archaea in green, Eukarya in blue, NCLDVs in red, other viruses and phages in pink and environmental sequences in black. For details on evolutionary models and phylogenetic methods, see Materials and Methods.
(TIF)

Figure S9 **DNAP B (DNA polymerase family B) phylogenetic tree (63 sequences, 100 positions).**
(TIF)

Figure S10 **PCNA (proliferating cell nuclear antigen) phylogenetic tree (41 sequences, 186 positions).**
(TIF)

Figure S11 **FEN (Flap endonuclease) phylogenetic tree (38 sequences, 123 positions).**
(TIF)

Figure S12 **RNAP II (RNA polymerase II) phylogenetic tree (81 sequences, 154 positions).**
(TIF)

Table S1 **Presence or absence of CroV ORFs assigned to one of the 47 NCVOGs corresponding to the reconstructed core gene set of the common ancestor of the NCLDV [6].** Footnote: This table is based on data from supplementary tables of reference [6], and of reference [19].
(DOCX)

Table S2 **Differences between CroV and Mimivirus regarding the presence/absence of CroV ORFs assigned to one of the 177 NCVOGs represented in two or more NCLDV families.** Footnote: This table is based on data from supplementary tables of reference [6], and of reference [19].
(DOCX)

Table S3 **Synteny between CroV and Mimivirus ORFs.**
(DOCX)

Table S4 **Presence/absence for COGs at least present in one NCLDV genome and that enable to distinguish between CroV and other NCLDVs.** Footnote: +, presence; −, absence; COGs functional categories are those defined in the COG database [42].
(DOCX)
