# Supplementary figures and images for: The Giant Cafeteria roenbergensis Virus That Infects a Widespread Marine Phagocytic Protist Is a New Member of the Fourth Domain of Life
Source: PLoS One. 2011 Apr 29;6(4):e18935. doi: 10.1371/journal.pone.0018935 (PMC3084725; doi:10.1371/journal.pone.0018935)

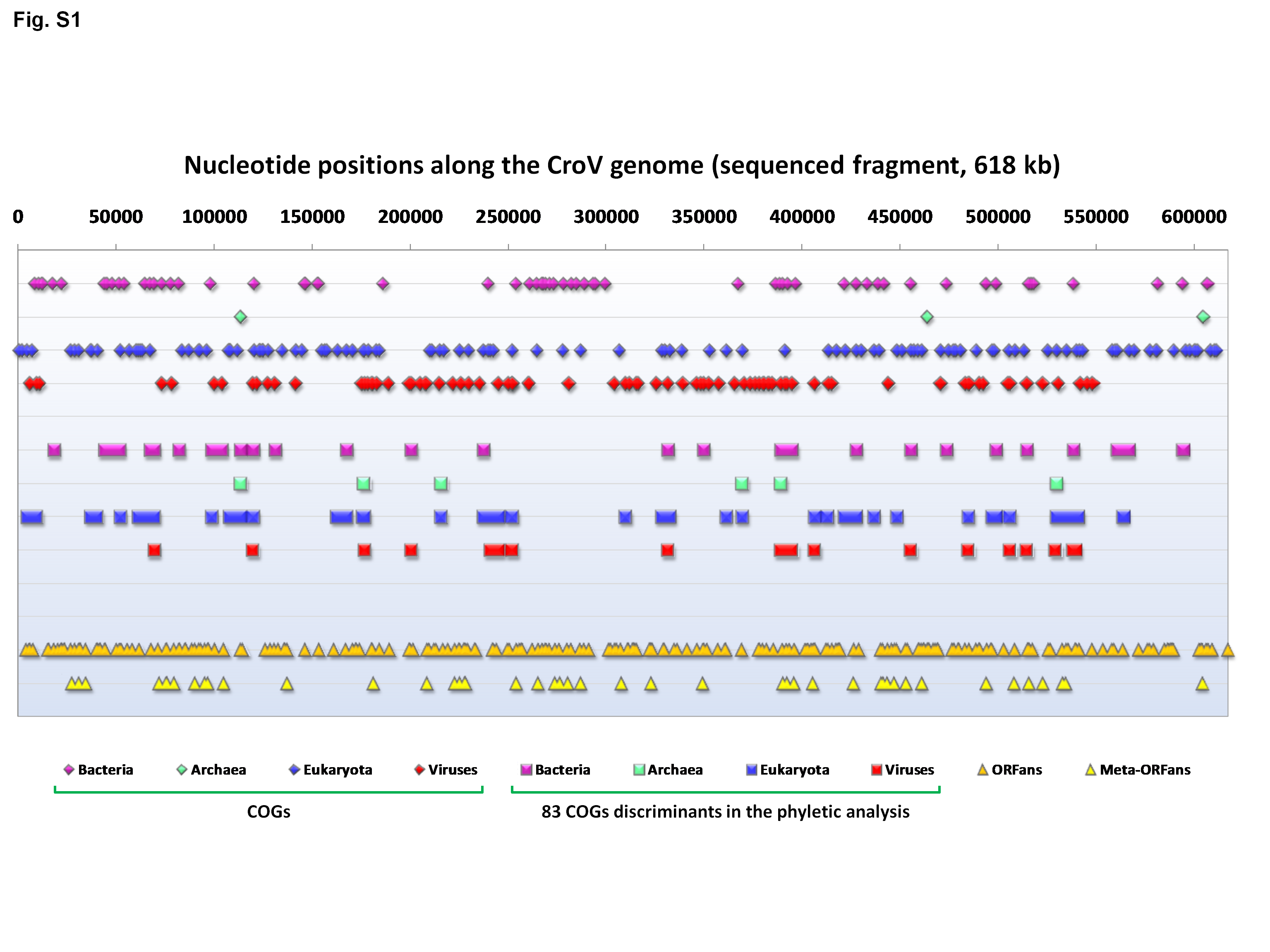

Supplement: Figure S1 — Genome map of the distribution along the CroV chromosome (sequenced fragment, 618 kb) of CroV ORFs assigned to COGs, and of ORFans and meta-ORFans. Taxonomy for the best BLASTp hits against the NCBI non-redundant protein sequence database are indicated for all CroV ORFs assigned to COGs, and for those corresponding to COGs found discriminant among NCLDVs in the phyletic analysis. (TIF) [file pone.0018935.s001.tif]

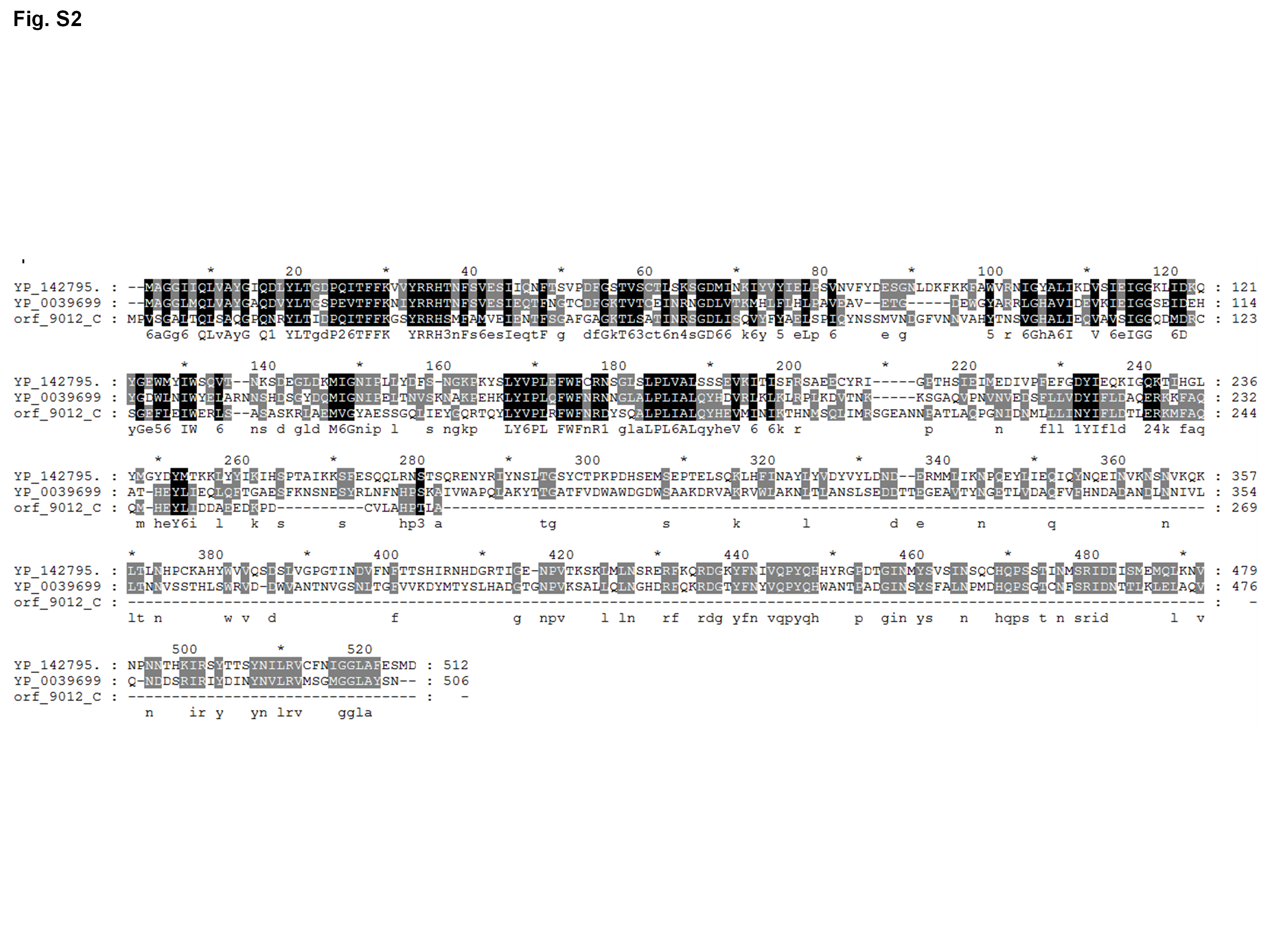

Supplement: Figure S2 — Alignment of amino acid sequences corresponding to the capsid protein of Acanthamoeba polyphaga Mimivirus (YP_142795.1; MIMI_R441), the major capsid protein of Cafeteria roenbergensis virus (YP_003969975.1; crov342), and Acanthamoeba castellanii . Schematic of the alignment was obtained using Genedoc [http://www.nrbsc.org/gfx/genedoc/]. (TIF) [file pone.0018935.s002.tif]

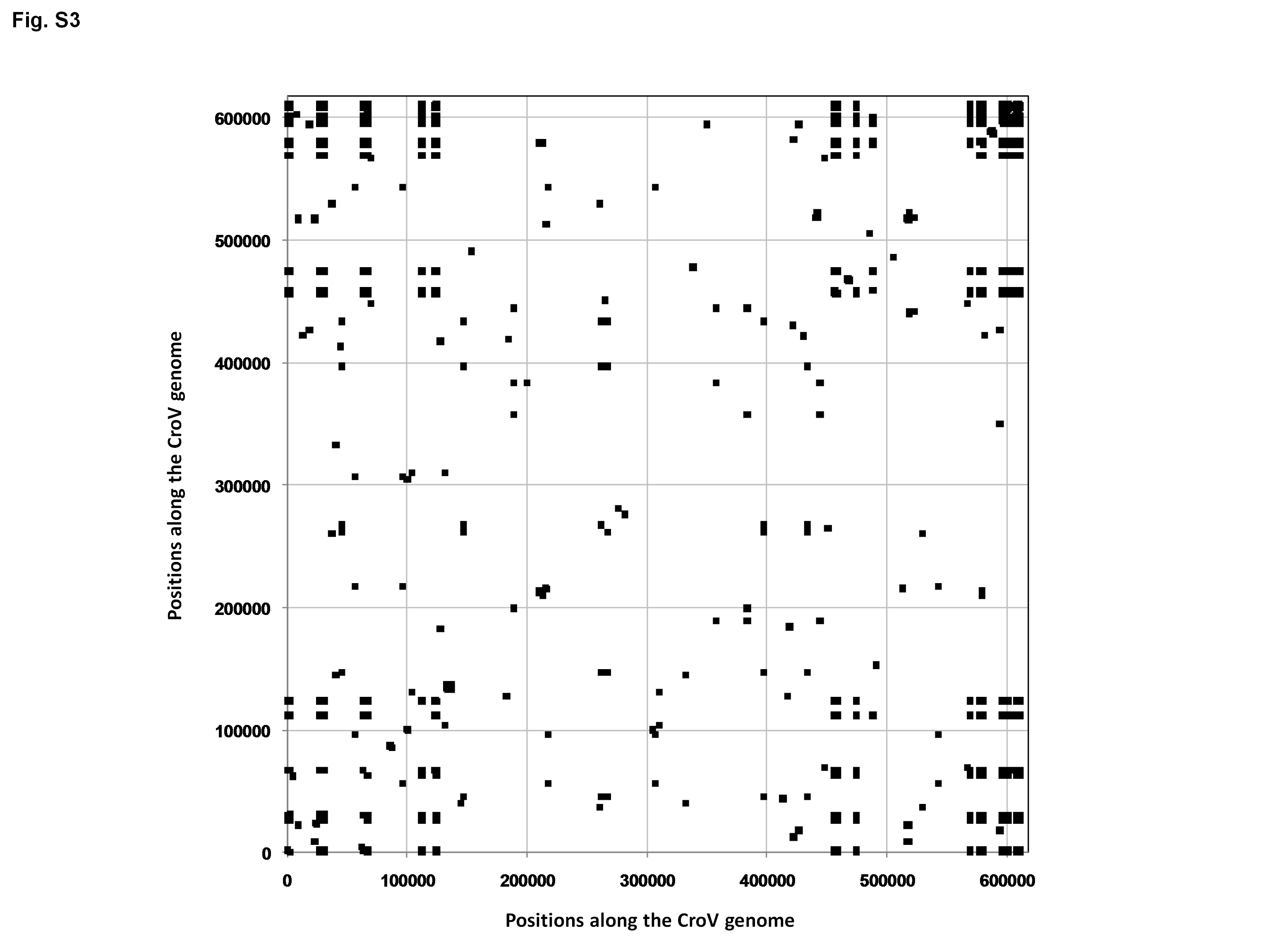

Supplement: Figure S3 — Dot plot showing positions of duplicated genes (CroV ORFs with significant BLASTp hits against the CroV proteome) on the CroV genome. (TIF) [file pone.0018935.s003.tif]

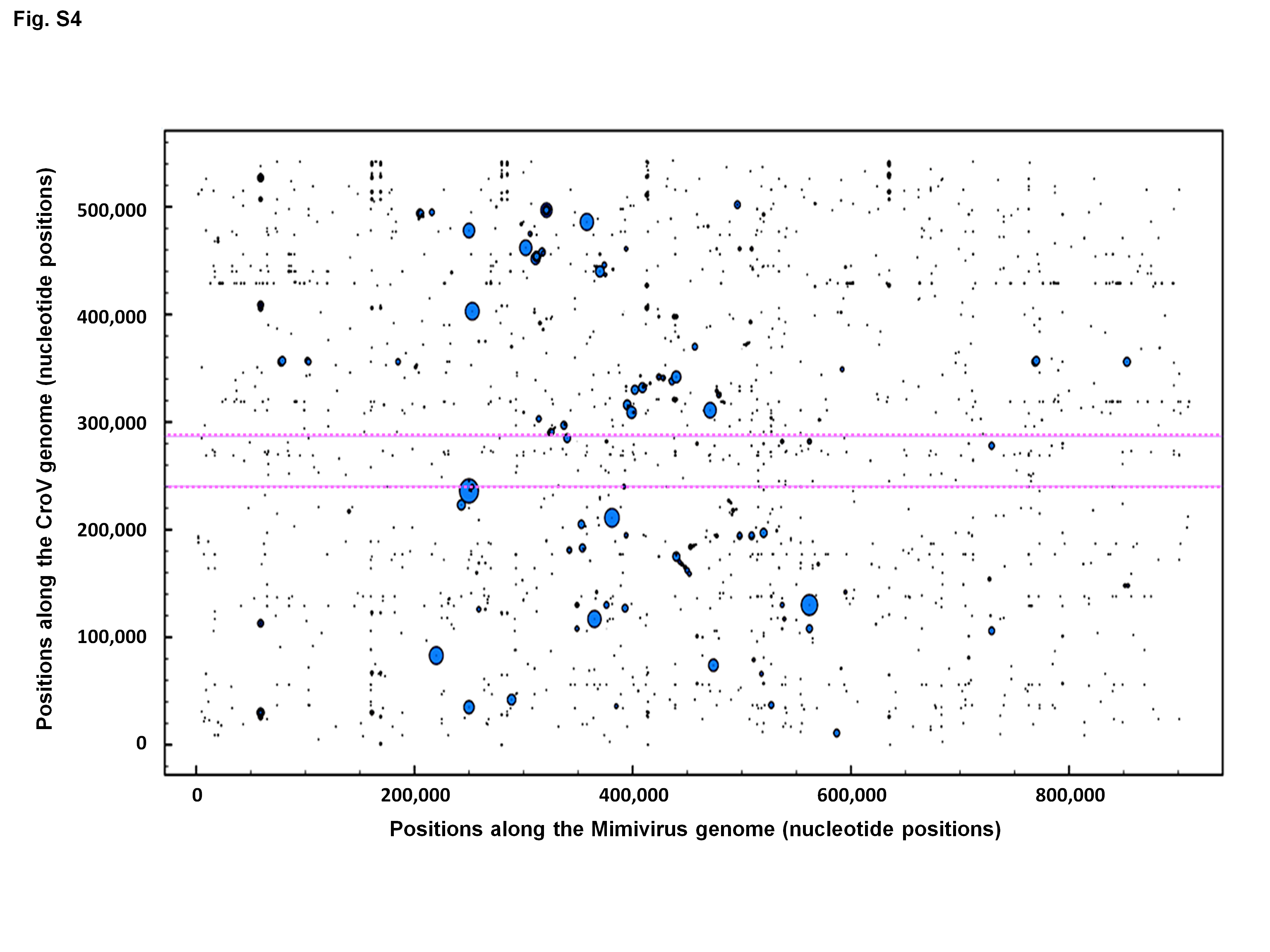

Supplement: Figure S4 — Dot plot of scores for reciprocal BLASTp hits (e-value<1e-4) between Mimivirus and CroV ORFs using bl2seq ( www.ncbi.nlm.nih.gov/blast/bl2seq/wblast2.cg ). Pink hachured lines delineate the region of the CroV genome of bacterial origin. (TIF) [file pone.0018935.s004.tif]

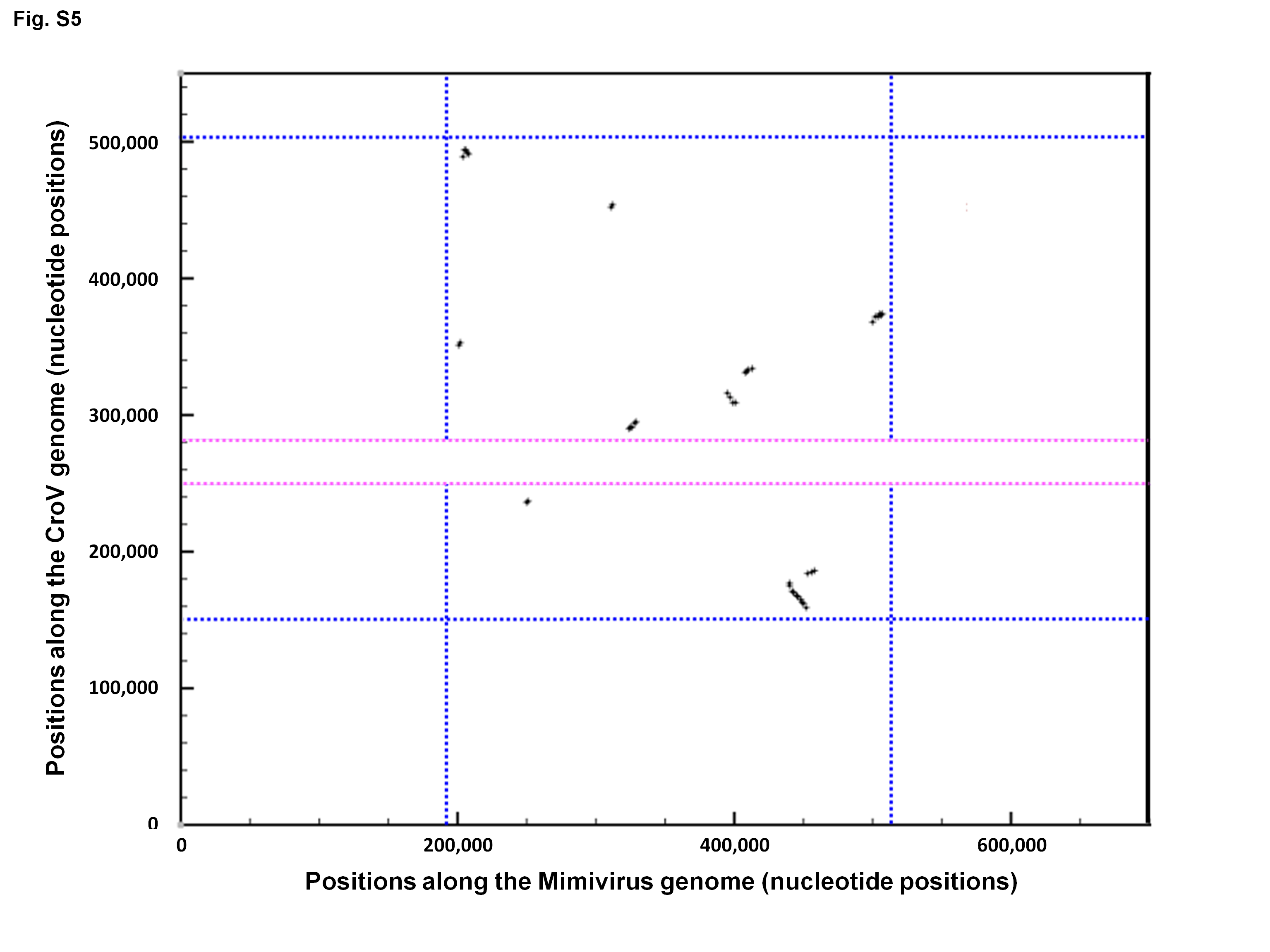

Supplement: Figure S5 — Dot plot of Mimivirus and CroV ORFs sharing reciprocal BLASTp hits (e-value<1e-100), as determined using bl2seq, and belonging to groups of at least two successive ORFs in synteny. Pink hachured lines delineate the region of the CroV genome of bacterial origin. (TIF) [file pone.0018935.s005.tif]

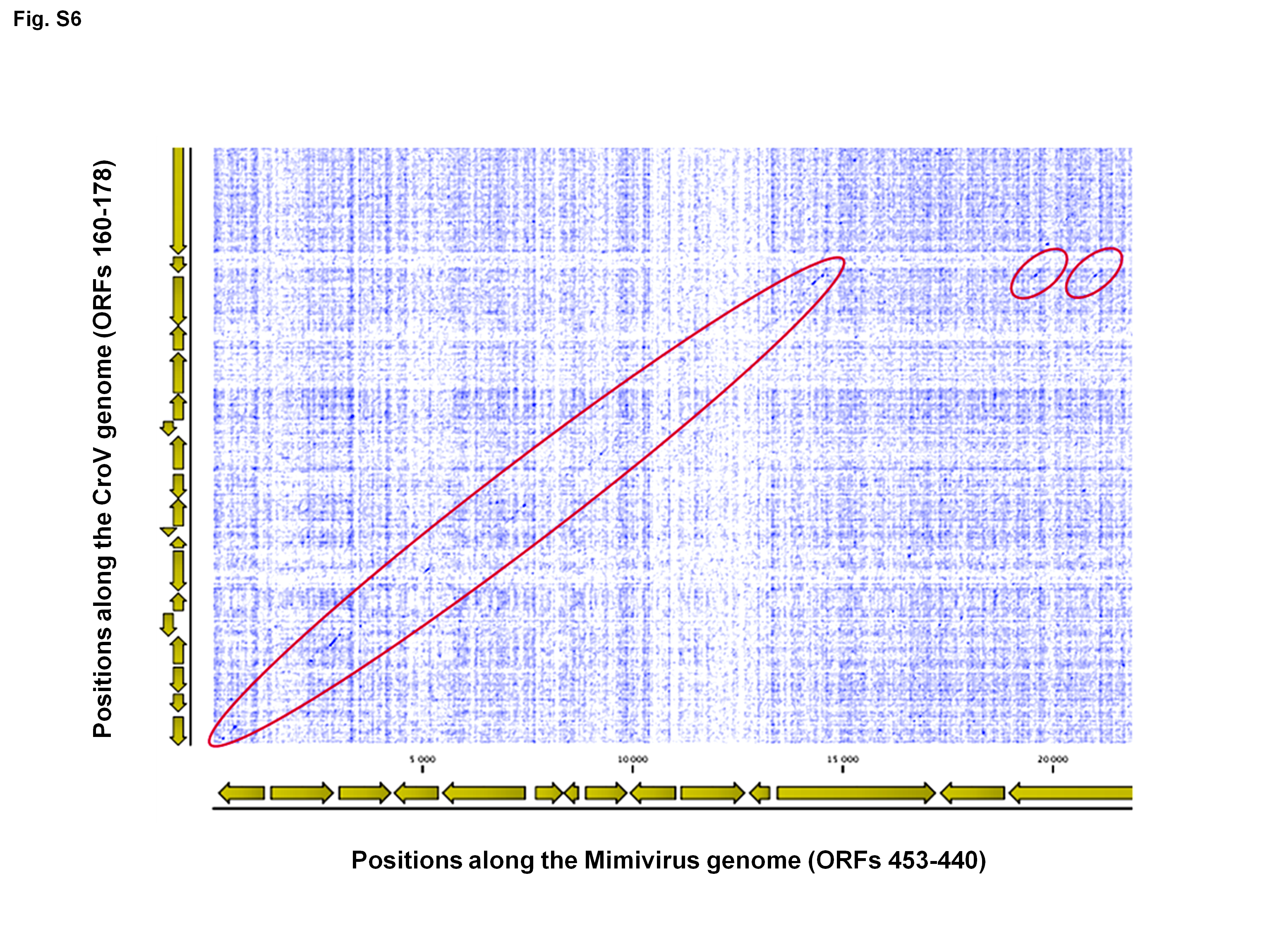

Supplement: Figure S6 — Nucleotide dot plot for windows of 30 nucleotides for regions corresponding to Mimivirus ORFs 453 to 440 and CroV ORFs 160 to 178. (TIF) [file pone.0018935.s006.tif]

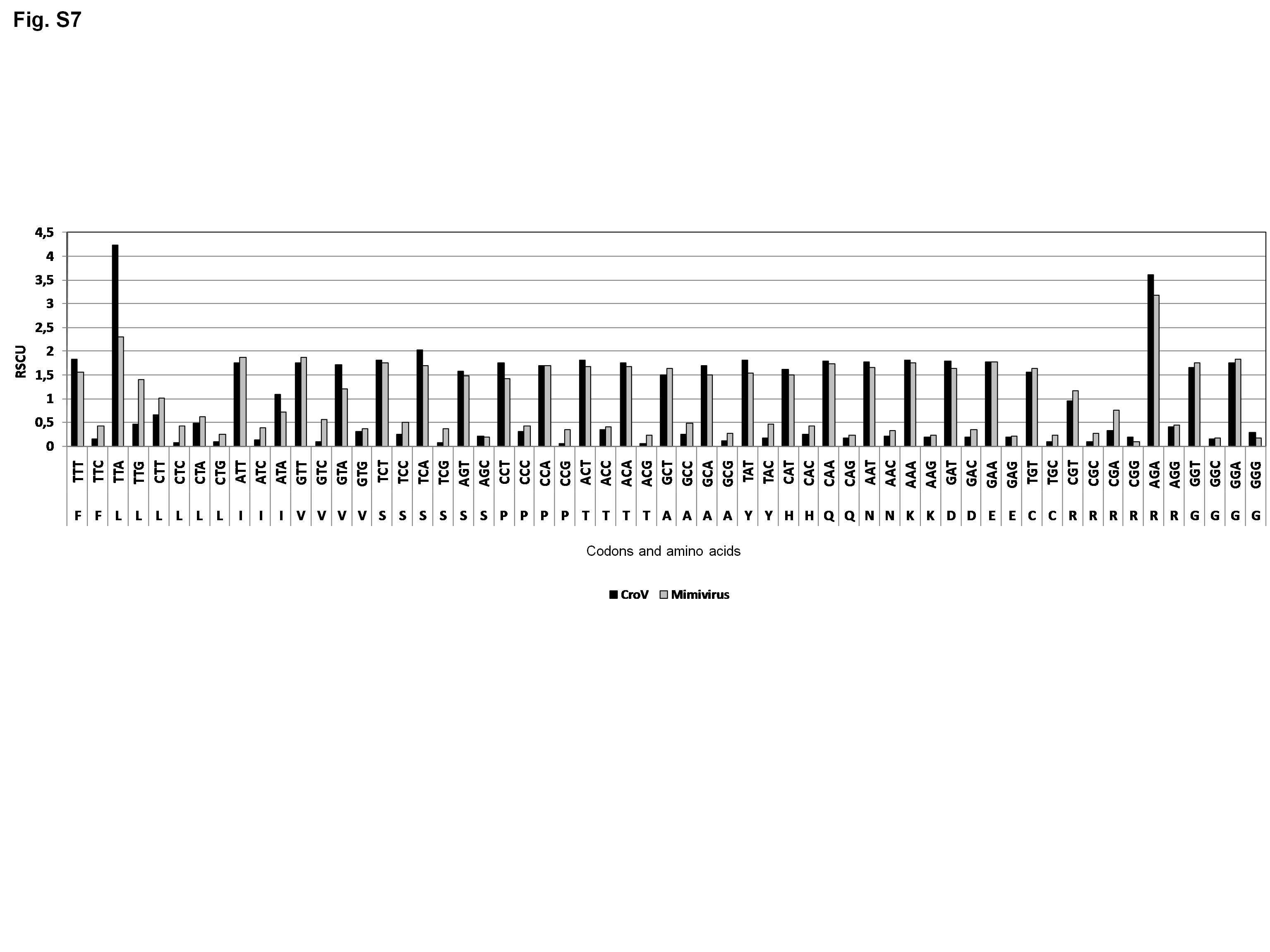

Supplement: Figure S7 — Relative synonymous codon usage (RSCU) for CroV and Mimivirus ORFs. (TIF) [file pone.0018935.s007.tif]

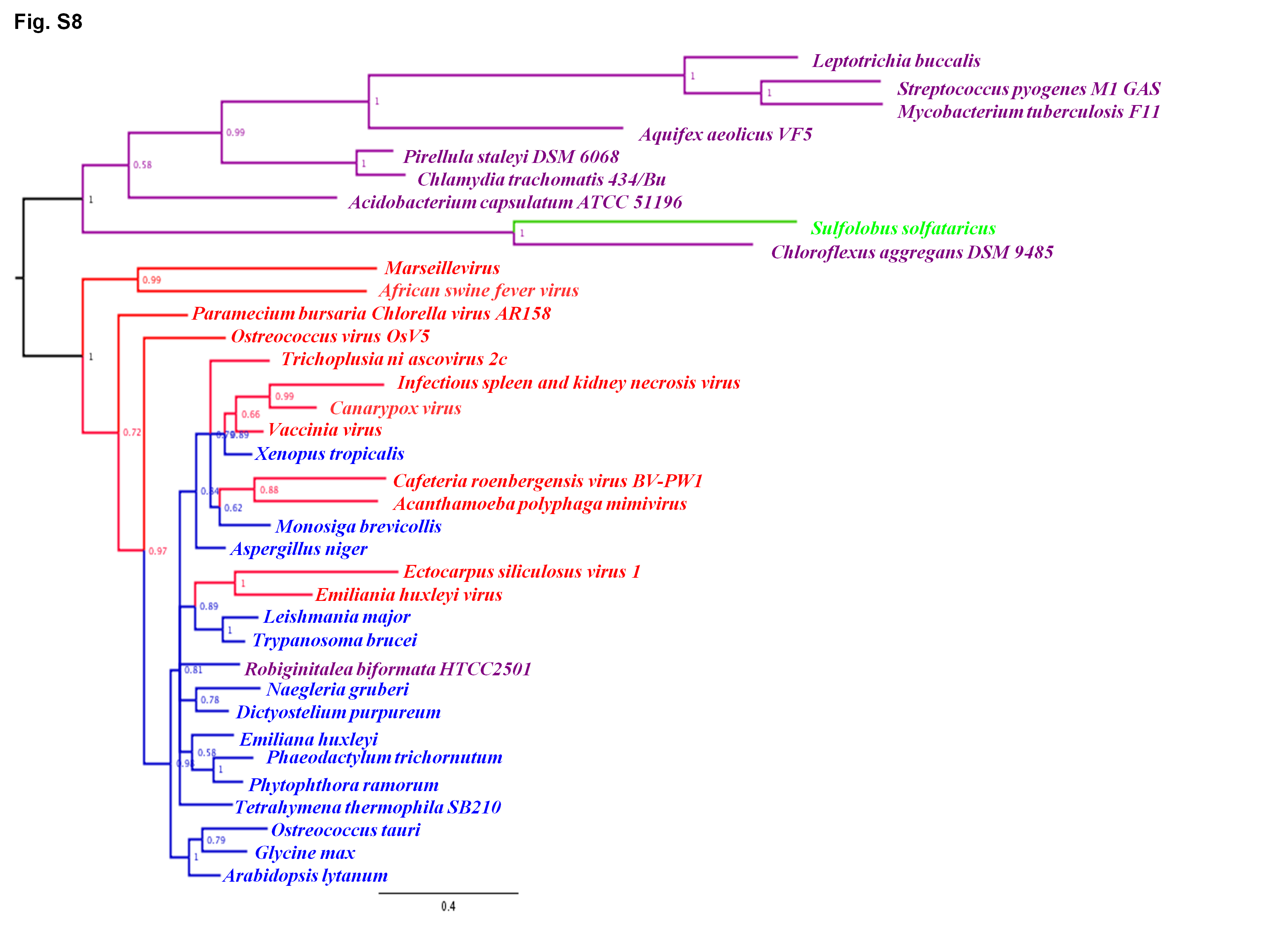

Supplement: Figure S8 — Phylogenetic trees constructed using a Bayesian approach are shown here and in Figures S9, S10, S11, and S12. This is an RNR (ribonucleotide reductase) phylogenetic tree (32 sequences, 214 positions). Bayesian posterior probabilities are mentioned near branches and are used as confidence values of tree branches. A color code was used to represent taxonomic groups, Bacteria in purple, Archaea in green, Eukarya in blue, NCLDVs in red, other viruses and phages in pink and environmental sequences in black. For details on evolutionary models and phylogenetic methods, see Materials and Methods. (TIF) [file pone.0018935.s008.tif]

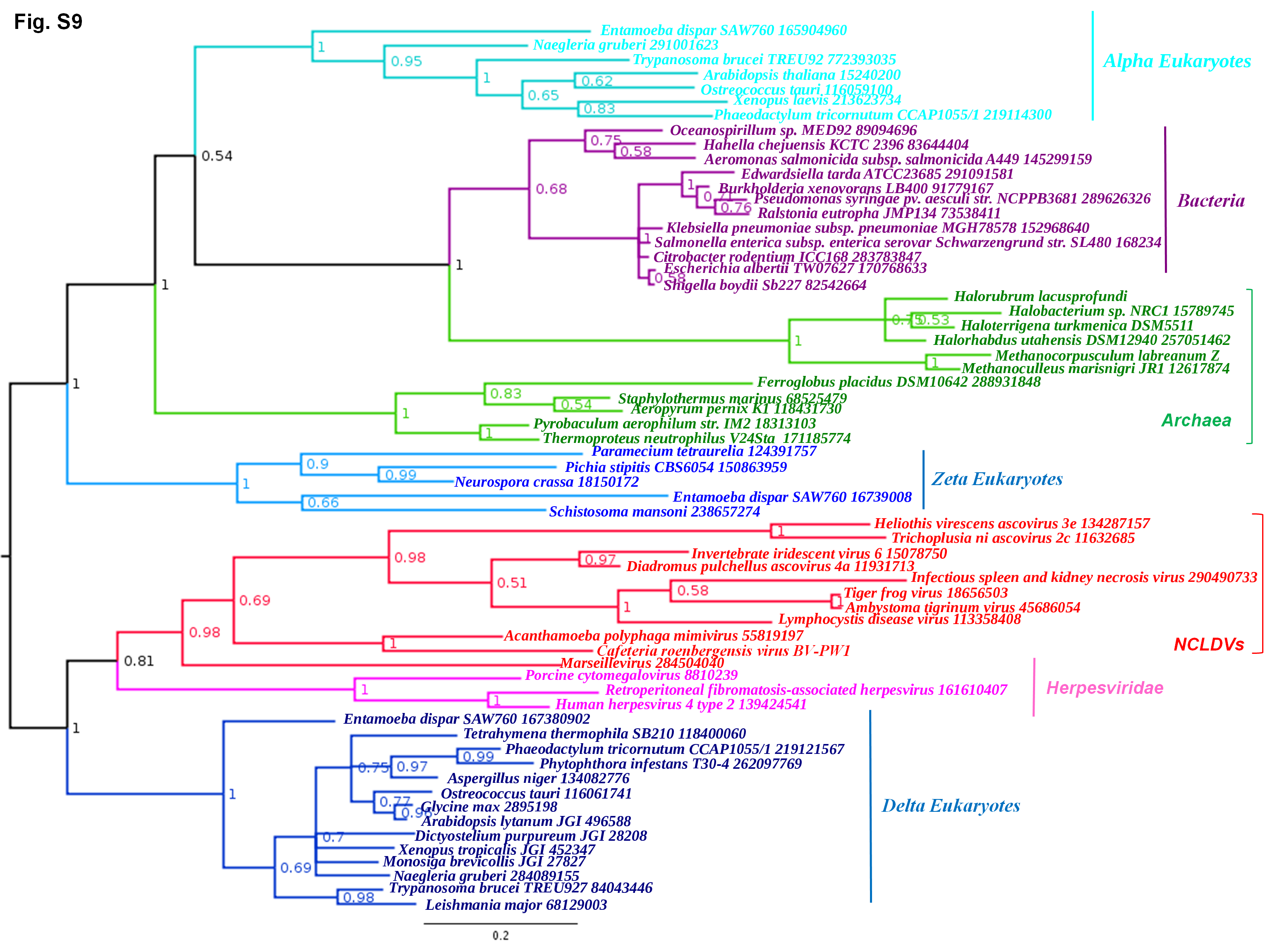

Supplement: Figure S9 — DNAP B (DNA polymerase family B) phylogenetic tree (63 sequences, 100 positions). (TIF) [file pone.0018935.s009.tif]

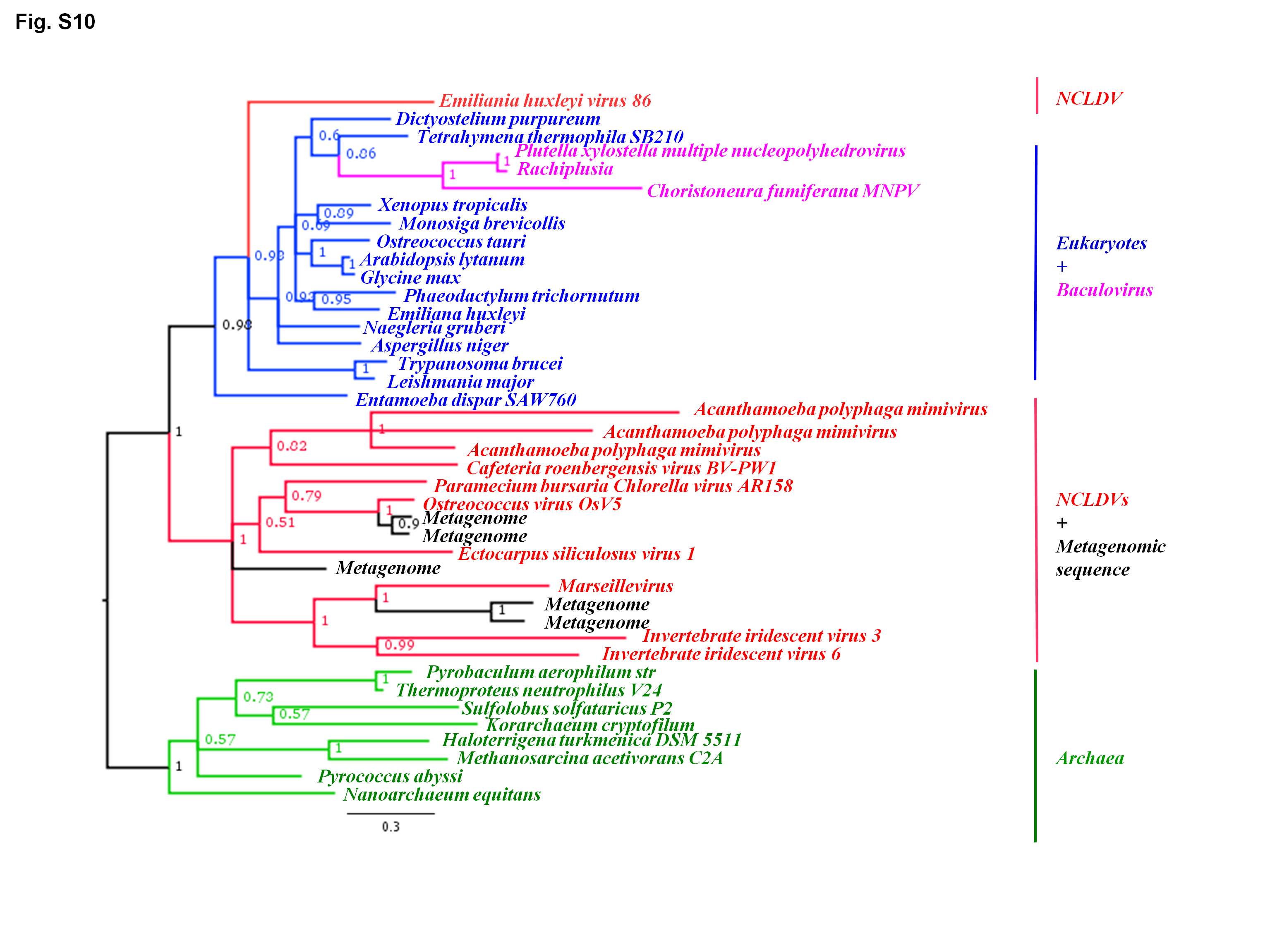

Supplement: Figure S10 — PCNA (proliferating cell nuclear antigen) phylogenetic tree (41 sequences, 186 positions). (TIF) [file pone.0018935.s010.tif]

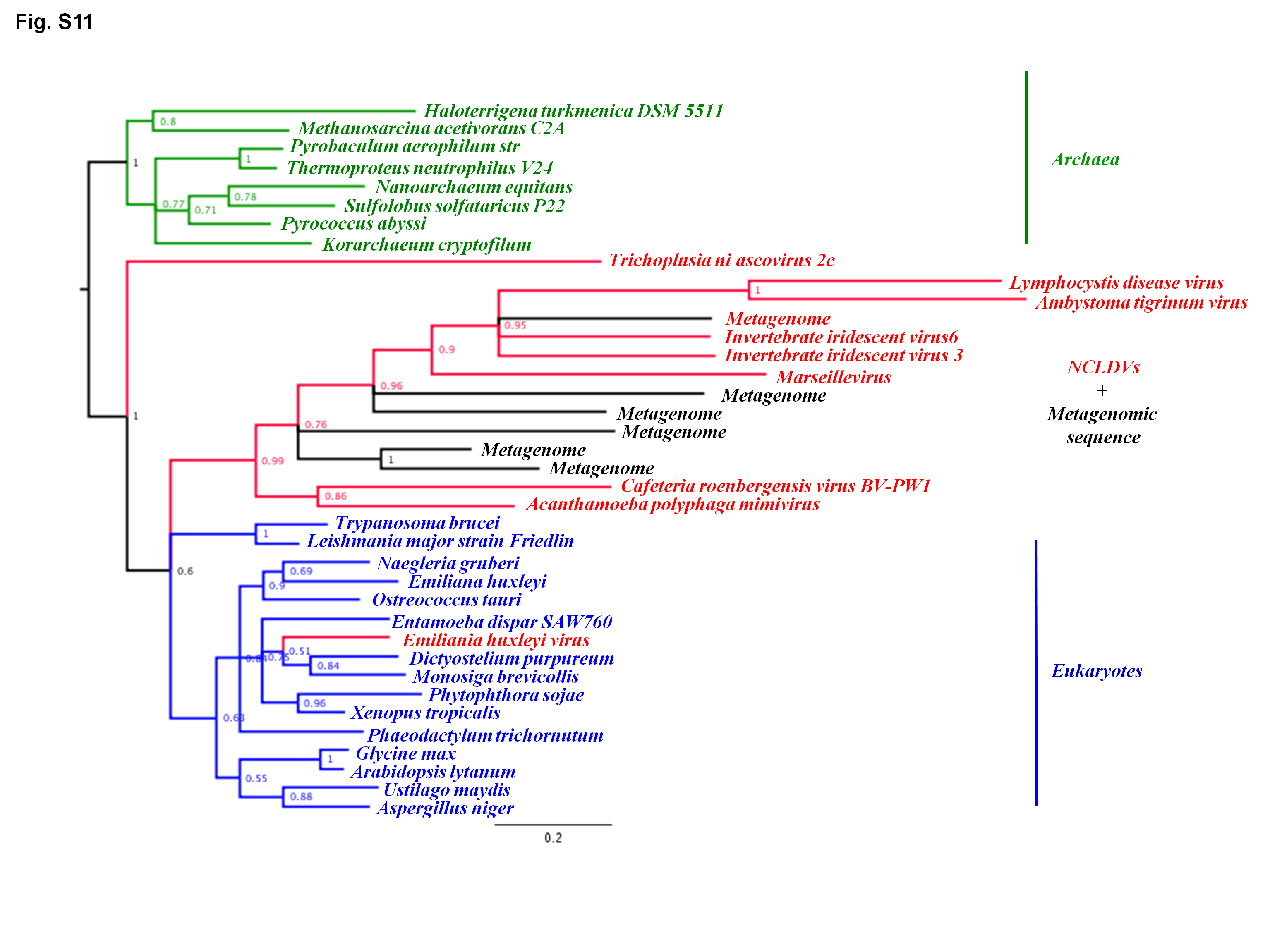

Supplement: Figure S11 — FEN (Flap endonuclease) phylogenetic tree (38 sequences, 123 positions). (TIF) [file pone.0018935.s011.tif]

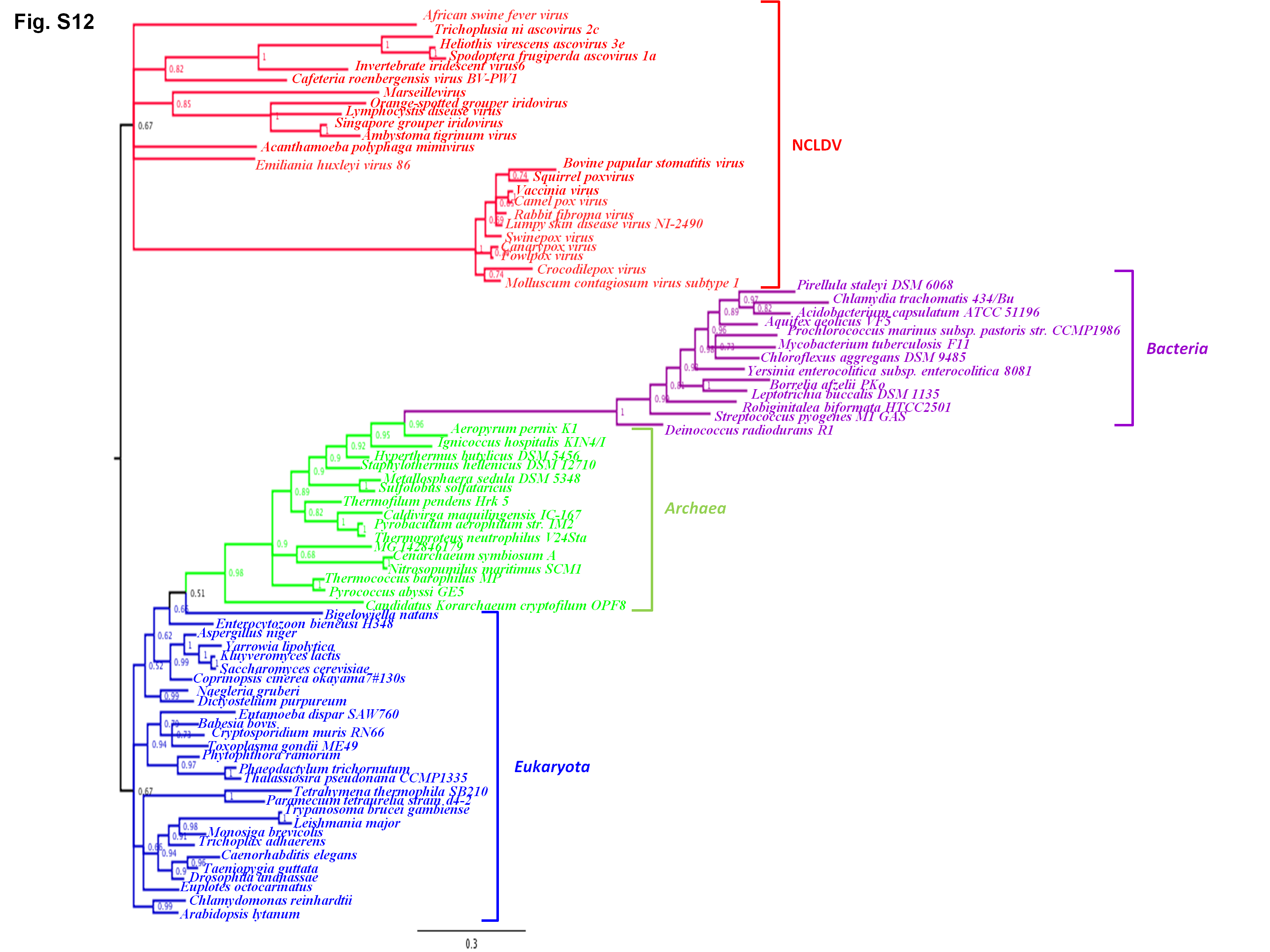

Supplement: Figure S12 — RNAP II (RNA polymerase II) phylogenetic tree (81 sequences, 154 positions). (TIF) [file pone.0018935.s012.tif]
