# Supplementary material for: The Giant Cafeteria roenbergensis Virus That Infects a Widespread Marine Phagocytic Protist Is a New Member of the Fourth Domain of Life
Source: PLoS One. 2011 Apr 29;6(4):e18935. doi: 10.1371/journal.pone.0018935 (PMC3084725; doi:10.1371/journal.pone.0018935)
Supplement: Table S1 — Presence or absence of CroV ORFs assigned to one of the 47 NCVOGs corresponding to the reconstructed core gene set of the common ancestor of the NCLDV [6]. Footnote: This table is based on data from supplementary tables of reference [6], and of reference [19]. (DOCX) [file pone.0018935.s013.docx]

**Table S1.** Differences between CroV and Mimivirus regarding the presence/absence of CroV ORFs assigned to one of the 177 NCVOGs represented in two or more NCLDV families

| **NCVOG id.** | **Functional category** | **Number of genomes present in a cluster** | | | | | | | **Cluster annotation** |
| --- | --- | --- | --- | --- | --- | --- | --- | --- | --- |
|  |  | *Poxviridae* | *Asfarviridae* | *Irido- and Ascoviridae* | *Phycodnaviridae* | Marseillevirus | *Mimiviridae* | CroV |  |
| NCVOG0002 | Miscellaneous | 1 | 0 | 0 | 5 | 0 | 2 | 0 | ABC transporter; pfam00005 |
| NCVOG0010 | Uncharacterized | 2 | 0 | 6 | 1 | 0 | 2 | 0 | pfam02498: Bro-N; BRO family, N-terminal domain: This family includes the N-terminus of baculovirus BRO and ALI motif proteins. |
| NCVOG0017 | Other metabolic functions | 2 | 0 | 0 | 1 | 0 | 2 | 0 | COG5092, NMT1, N-myristoyl transferase (pfam01233, pfam02799) |
| NCVOG0021 | Uncharacterized | 0 | 0 | 4 | 0 | 0 | 2 | 0 | collagen triple helix repeat containing protein (pfam01391) |
| NCVOG0036 | DNA replication, recombination and repair | 20 | 0 | 0 | 1 | 0 | 2 | 0 | DNA topoisomerase I |
| NCVOG0040 | Other metabolic functions | 19 | 0 | 3 | 6 | 0 | 2 | 0 | cd00127, DSPc, Dual specificity phosphatases (DSP); Ser/Thr and Tyr protein phosphatases |
| NCVOG0050 | Other metabolic functions | 4 | 0 | 2 | 5 | 0 | 2 | 0 | Esterase lipase superfamily (cl09107, pfam07859, pfam00135, COG0400) |
| NCVOG0062 | DNA replication, recombination and repair | 0 | 0 | 4 | 7 | 0 | 2 | 0 | GIY-YIG-like endonuclease (pfam01541) |
| NCVOG0070 | Other metabolic functions | 0 | 0 | 0 | 1 | 0 | 2 | 0 | Vacuolar (H+)-ATPase G subunit (pfam03179) |
| NCVOG0075 | Transcription and RNA processing | 0 | 0 | 0 | 1 | 0 | 2 | 0 | Helix-turn-helix XRE-family like proteins (pfam01381, cd00093) |
| NCVOG0199 | DNA replication, recombination and repair | 0 | 0 | 0 | 6 | 0 | 2 | 0 | uncharacterized protein |
| NCVOG0211 | Virion structure and morphogenesis | 20 | 1 | 11 | 0 | 0 | 2 | 0 | myristylated IMV envelope protein (pfam02442: Lipid membrane protein of large eukaryotic DNA viruses) |
| NCVOG0229 | Uncharacterized | 0 | 0 | 4 | 2 | 0 | 2 | 0 | uncharacterized repeats |
| NCVOG0279 | Other metabolic functions | 0 | 0 | 0 | 8 | 0 | 2 | 0 | Putative methyl transferase (smart00317) |
| NCVOG0310 | Other metabolic functions | 14 | 0 | 0 | 0 | 0 | 2 | 0 | Serpin (serine protease inhibitor); pfam00079, cd00172 |
| NCVOG0318 | Other metabolic functions | 2 | 0 | 0 | 6 | 0 | 2 | 0 | Thioredoxin-like proteins of Phycodnaviridae; in q3_Ectsi13242599 it was upstream to cd01049 domain (Ribonucleotide Reductase, R2/beta subunit (RNRR2)) |
| NCVOG0575 | Uncharacterized | 0 | 1 | 0 | 0 | 0 | 2 | 0 | hypothetical protein |
| NCVOG0773 | Uncharacterized | 0 | 0 | 2 | 0 | 0 | 2 | 0 | hypotetical protein of mama-, mimiviruses |
| NCVOG1046 | Miscellaneous | 0 | 0 | 0 | 4 | 0 | 2 | 0 | Zn-finger - containing protein |
| NCVOG1051 | Uncharacterized | 0 | 0 | 3 | 0 | 0 | 2 | 0 | hypothetical protein conserved in ascovirus, mimi-, mamavirus |
| NCVOG1122 | Virion structure and morphogenesis | 20 | 0 | 9 | 0 | 0 | 2 | 0 | Myristylated protein; pfam03003, DUF230 |
| NCVOG1125 | Uncharacterized | 0 | 0 | 0 | 3 | 0 | 2 | 0 | protein of unknown function DUF305 conserved in bacteria; found in mimi-, mama-, Chloroviruses |
| NCVOG1130 | Uncharacterized | 0 | 0 | 0 | 1 | 0 | 2 | 0 | membrane protein with eukaryotic homologs (pfam03798: TRAM_LAG1_CLN8, TLC domain) |
| **NCVOG id.** | **Functional category** | **Number of genomes present in a cluster** | | | | | | | **Cluster annotation** |
|  |  | *Poxviridae* | *Asfarviridae* | *Irido- and Ascoviridae* | *Phycodnaviridae* | Marseillevirus | *Mimiviridae* | CroV |  |
| NCVOG1152 | Transcription and RNA processing | 20 | 0 | 0 | 0 | 0 | 1 | 0 | poly(A) polymerase small subunit PAPS |
| NCVOG1342 | Uncharacterized | 0 | 0 | 0 | 8 | 0 | 2 | 0 | hypothetical mimi-, mama-, Phycodnavirus protein |
| NCVOG1360 | Miscellaneous | 15 | 0 | 1 | 0 | 0 | 2 | 0 | KilA domain (pfam04383); always is present at N-terminal except for mimiviruses. Sometimes is followed by a RING-finger domain |
| NCVOG1424 | Uncharacterized | 3 | 0 | 1 | 0 | 0 | 2 | 0 | uncharacterized domain; found downstream KilA, BRO, and MSV199 domains. Also is found in some baculoviruses (gi 165969059, 18138388) |
| NCVOG0024 | DNA replication, recombination and repair | 0 | 1 | 0 | 0 | 1 | 2 | 0 | Superfamily II helicase related to herpesvirus replicative helicase (origin-binding protein UL9), pfam03121 |
| NCVOG0058 | Other metabolic functions | 0 | 0 | 0 | 6 | 1 | 2 | 0 | Flavin-containing amine oxidoreductase conserved in mama-, mimi, Marseille virus, and Chloroviridae |
| NCVOG0064 | Translation | 0 | 0 | 0 | 0 | 1 | 2 | 0 | GTP binding translation elongation factor |
| NCVOG0248 | DNA replication, recombination and repair | 0 | 0 | 5 | 1 | 1 | 2 | 0 | Pif1 helicase |
| NCVOG0305 | Signal transduction regulation | 0 | 0 | 0 | 0 | 1 | 2 | 0 | Mimi-, mama-, Marseille virus tandem-duplicated ST (or Tyrosine) kinase; apparent eukaryotic origin (gi\|66804679: protein kinase, TKL group [Dictyostelium discoideum AX4]) |
| NCVOG0308 | DNA replication, recombination and repair | 0 | 0 | 5 | 0 | 1 | 2 | 0 | DNA repair exonuclease (COG0419,COG0420) |
| NCVOG0312 | DNA replication, recombination and repair | 0 | 0 | 5 | 0 | 1 | 2 | 0 | COG1196, Smc, Chromosome segregation ATPases |
| NCVOG0314 | Translation | 0 | 0 | 0 | 0 | 1 | 2 | 0 | Translation initiation factor SUI1 |
| NCVOG0349 | Miscellaneous | 0 | 0 | 0 | 0 | 1 | 2 | 0 | Zn-finger containing protein; similar to EGF-like domain containing protein |
| NCVOG0351 | Uncharacterized | 0 | 0 | 0 | 0 | 1 | 2 | 0 | cell division cycle 123 homolog |
| NCVOG0352 | Other metabolic functions | 0 | 0 | 0 | 0 | 1 | 2 | 0 | Metal dependent phosphohydrolase with conserved 'HD' motif |
| NCVOG0435 | DNA replication, recombination and repair | 0 | 0 | 0 | 1 | 1 | 2 | 0 | AlkB, Alkylated DNA repair protein unique in NCLDV |
| NCVOG0436 | Uncharacterized | 0 | 0 | 0 | 0 | 1 | 2 | 0 | short hypothetical protein |
| NCVOG0480 | Other metabolic functions | 0 | 0 | 0 | 0 | 1 | 2 | 0 | pfam01368, DHH, DHH family phosphohydrolase |
| NCVOG0507 | Uncharacterized | 0 | 0 | 0 | 0 | 1 | 2 | 0 | hypothetical protein of Marseille virus, mama-, mimivirus. |
| NCVOG0632 | Uncharacterized | 0 | 0 | 0 | 9 | 1 | 2 | 0 | conserved hypotetical protein |
| NCVOG0716 | Uncharacterized | 0 | 0 | 0 | 0 | 1 | 2 | 0 | P-loop ATPase or GTPase |
| NCVOG0772 | Uncharacterized | 0 | 0 | 0 | 0 | 1 | 2 | 0 | Uncharacterized protein conserved in bacteria, Marseille virus, mama- and mimivirus |
| NCVOG0815 | Uncharacterized | 0 | 0 | 0 | 0 | 1 | 2 | 0 | Uncharacterized protein conserved in bacteria, Marseille virus, mama- and mimivirus |
| **NCVOG id.** | **Functional category** | **Number of genomes present in a cluster** | | | | | | | **Cluster annotation** |
|  |  | *Poxviridae* | *Asfarviridae* | *Irido- and Ascoviridae* | *Phycodnaviridae* | Marseillevirus | *Mimiviridae* | CroV |  |
| NCVOG0979 | Translation | 0 | 0 | 0 | 0 | 1 | 2 | 0 | Eukaryotic peptide chain release (translation termination) factor 1 |
| NCVOG1044 | Miscellaneous | 0 | 0 | 0 | 0 | 1 | 2 | 0 | WD-repeat family proteins (pfam00400) |
| NCVOG1074 | Host-virus interactions | 0 | 0 | 10 | 0 | 1 | 2 | 0 | restriction-fold endonuclease; D...H...D conserved. |
| NCVOG1087 | Other metabolic functions | 0 | 0 | 7 | 2 | 1 | 2 | 0 | papain-like cysteine peptidase (Cathepsin B group) |
| NCVOG1115 | Other metabolic functions | 20 | 0 | 0 | 0 | 1 | 2 | 0 | uracil-DNA glycosylase |
| NCVOG1128 | Other metabolic functions | 0 | 0 | 0 | 0 | 1 | 2 | 0 | glycosyl transferase |
| NCVOG1154 | Host-virus interactions | 0 | 0 | 2 | 3 | 1 | 2 | 0 | SWIB/MDM2 domain-containing protein (chromatin condensation) |
| NCVOG1358 | Miscellaneous | 11 | 0 | 0 | 0 | 1 | 2 | 0 | pfam00651, BTB/POZ domain |
| NCVOG1361 | Uncharacterized | 2 | 1 | 4 | 1 | 1 | 2 | 0 | pfam10544, T5orf172 domain |
| NCVOG1068 | Nucleotide metabolism | 17 | 1 | 4 | 8 | 0 | 0 | crov069 | dUTPase (cl00493) |
| NCVOG0232 | DNA replication, recombination and repair | 0 | 0 | 0 | 4 | 1 | 0 | crov347 | D12 class N6 adenine-specific DNA methyltransferase (pfam02086) |
| NCVOG0333 | Other metabolic functions | 3 | 0 | 1 | 2 | 1 | 0 | crov350 | Ubiquitin |
| NCVOG1088 | Transcription and RNA processing | 0 | 1 | 11 | 0 | 1 | 0 | crov480 | RNA ligase (conserved in irido-, asfa- asco- and Marseille viruses) |

This table is based on data from supplementary tables of reference [6], and of reference [19]
