# Supplementary material for: The Giant Cafeteria roenbergensis Virus That Infects a Widespread Marine Phagocytic Protist Is a New Member of the Fourth Domain of Life
Source: PLoS One. 2011 Apr 29;6(4):e18935. doi: 10.1371/journal.pone.0018935 (PMC3084725; doi:10.1371/journal.pone.0018935)
Supplement: Table S2 — Differences between CroV and Mimivirus regarding the presence/absence of CroV ORFs assigned to one of the 177 NCVOGs represented in two or more NCLDV families. Footnote: This table is based on data from supplementary tables of reference [6], and of reference [19]. (DOCX) [file pone.0018935.s014.docx]

**Table S2.** Synteny between CroV and Mimivirus ORFs.

| **CroV ORFs** | **Predicted function** | **Mimivirus ORFs** |
| --- | --- | --- |
|  |  |  |
| crov160 | TATA-box binding protein | MIMI_R453 |
| crov163 | Holliday junction resolvase | MIMI_L451 |
| crov164 | transcription factor | MIMI_R450 |
| crov166 | unknown | MIMI_R449 |
| crov168 | unknown | MIMI_R447 |
| crov169 | papatin-like phospholipase | MIMI_L446 |
| crov170 | contains DnaJ/Hsp40 domain |  |
| crov171 | ADP-ribosyl glycohydrolase | MIMI_L444 |
| crov172 | protein disulfide isomerase | MIMI_R443 |
| crov176 | capsid protein 4 | MIMI_R441 |
|  |  |  |
|  |  |  |
| crov185 | unknown | MIMI_L454 |
| crov186 | unknown | MIMI_R457 |
| crov187 | unknown | MIMI_R459 |
|  |  |  |
|  |  |  |
| crov230 | transcription initiation factor TFIIB | MIMI_L250 |
| crov237 | Lon protease | MIMI_L251 |
| crov238 | unknown | MIMI_R252 |
|  |  |  |
|  |  |  |
| crov291 | metal-dependent hydrolase | MIMI_R325 |
| crov292 | VV A7-like early transcription factor LSU | MIMI_R327 |
| crov295 | unknown | MIMI_R329 |
| crov296 | unknown | MIMI_L330 |
|  |  |  |
| crov298 | unknown | MIMI_R338 |
| crov299 | transcription elongation factor TFIIS | MIMI_R339 |
|  |  |  |
|  |  |  |
| crov313 | phosphoesterase | MIMI_R398 |
| crov316 | VV A18-like helicase | MIMI_L396 |
|  |  |  |
| crov318 | unknown | MIMI_L485 |
| crov319 | contains ankyrin repeats | MIMI_L484 |
|  |  |  |
| crov325 | DNA topoisomerase IIA, intein-containing | MIMI_R480 |
| crov326 | unknown | MIMI_R481 |
|  |  |  |
| crov331 | unknown | MIMI_R409 |
| crov332 | major core protein | MIMI_L410 |
| crov333 | unknown | MIMI_R411 |
| crov334 | unknown | MIMI_L414 |
|  |  |  |
|  |  |  |
| crov351 | unknown | MIMI_L202 |
| crov353 | unknown | MIMI_R203 |
|  |  |  |
| crov368 | Rpb1, DNA-directed RNA polymerase II subunit 1 | MIMI_R501 |
| crov372 | unknown | MIMI_R505 |
| crov373 | unknown | MIMI_L507 |
| crov374 | unknown | MIMI_R508 |
|  |  |  |
| crov452 | ribonucleoside-dipohosphate reductase small subunit | MIMI_L312 |
| crov454 | ribonucleoside-diphosphate reductase LSU, intein-containing | MIMI_R313 |
|  |  |  |
| crov457 | ubiquitin carboxyl-terminal hydrolase | MIMI_R319 |
| crov458 | family X DNA polymerase | MIMI_L318 |
|  |  |  |
| crov474 | unknown | MIMI_L308 |
| crov475 | family 2C Ser/Thr phosphatase | MIMI_R307 |
|  |  |  |
|  |  |  |
| crov489 | Ser/Thr protein kinase | MIMI_L205 |
| crov491 | Rpb6, DNA-directed RNA polymerase II subunit 6 | MIMI_R209 |
| crov492 | Rpb9, DNA-directed RNA polymerase II subunit 9 | MIMI_L208 |
| crov494 | VV D5-like primase/helicase | MIMI_L206 |
|  |  |  |
