# Supplementary material for: The Giant Cafeteria roenbergensis Virus That Infects a Widespread Marine Phagocytic Protist Is a New Member of the Fourth Domain of Life
Source: PLoS One. 2011 Apr 29;6(4):e18935. doi: 10.1371/journal.pone.0018935 (PMC3084725; doi:10.1371/journal.pone.0018935)
Supplement: Table S3 — Synteny between CroV and Mimivirus ORFs. (DOCX) [file pone.0018935.s015.docx]

**Table S3.** Presence or absence of CroV ORFs assigned to one of the 47 NCVOGs corresponding to the reconstructed core gene set of the common ancestor of the NCLDV [6]

| **NCVOG id.** | **NCVOGs including proteins from all 45 analyzed viruses** | **Functional category** | **Number of genomes present in a cluster** | | | | | | | **NCVOG annotation** |
| --- | --- | --- | --- | --- | --- | --- | --- | --- | --- | --- |
|  |  |  | *Poxviridae* | *Asfarviridae* | *Irido- and Ascoviridae* | *Phycodnaviridae* | Marseillevirus | *Mimiviridae* | CroV |  |
| **NCVOG0022** | Yes | Virion structure and morphogenesis | 20/20 | 1/1 | 11/11 | 10/10 | 1/1 | 2/2 | **crov176** | NCLDV major capsid protein (pfam03340 for Poxviridae; pfam04451 for others) |
| **NCVOG0023** | Yes | DNA replication, recombination and repair | 20/20 | 1/1 | 11/11 | 10/10 | 1/1 | 2/2 | **crov494** | D5-like helicase-primase |
| **NCVOG0038** | Yes | DNA replication, recombination and repair | 20/20 | 1/1 | 11/11 | 10/10 | 1/1 | 2/2 | **crov497** | DNA polymerase elongation subunit family B |
| **NCVOG0249** | Yes | Virion structure and morphogenesis | 20/20 | 1/1 | 11/11 | 10/10 | 1/1 | 2/2 | **crov338** | A32-like packaging ATPase |
| **NCVOG0262** | Yes | Transcription and RNA processing | 20/20 | 1/1 | 11/11 | 10/10 | 1/1 | 2/2 | **crov341** | pfam04947, Poxvirus Late Transcription Factor VLTF3 like |
|  |  |  |  |  |  |  |  |  |  |  |
| **NCVOG0004** | **-** | DNA replication, recombination and repair | 2/20 | 1/1 | 0/11 | 0/10 | 1/1 | 2/2 | **crov458** | AP (apurinic) endonuclease family 2 - bacterial |
| **NCVOG0037** | **-** | DNA replication, recombination and repair | 1/20 | 1/1 | 2/11 | 8/10 | 1/1 | 2/2 | **crov325** | DNA topoisomerase II |
| **NCVOG0052** | **-** | Virion structure and morphogenesis | 20/20 | 1/1 | 11/11 | 9/10 | 1/1 | 2/2 | **crov143** | Disulfide (thiol) oxidoreductase; Erv1 / Alr family (pfam04777) |
| **NCVOG0076** | **-** | DNA replication, recombination and repair | 20/20 | 1/1 | 5/11 | 9/10 | 1/1 | 2/2 | **crov316** | DNA or RNA helicases of superfamily II (COG1061) |
| **NCVOG0236** | **-** | Transcription and RNA processing | 20/20 | 1/1 | 4/11 | 1/10 | 1/1 | 2/2 | **crov446** | Nudix hydrolase (D10 ortholog) |
| **NCVOG0261** | **-** | Transcription and RNA processing | 20/20 | 1/1 | 11/11 | 0/10 | 1/1 | 2/2 | **crov292** | Poxvirus early transcription factor (VETF), large subunit (pfam04441) |
| **NCVOG0271** | **-** | Transcription and RNA processing | 20/20 | 1/1 | 11/11 | 1/10 | 1/1 | 2/2 | **crov224** | DNA-directed RNA polymerase subunit beta |
| **NCVOG0272** | **-** | Transcription and RNA processing | 18/20 | 1/1 | 9/11 | 8/10 | 1/1 | 2/2 | **crov299** | Transcription factor S-II (TFIIS)-domain-containing protein |
| **NCVOG0273** | **-** | Transcription and RNA processing | 0/20 | 1/1 | 10/11 | 1/10 | 1/1 | 2/2 | **crov439** | Divergent DNA-directed RNA polymerase subunit 5 |
| **NCVOG0274** | **-** | Transcription and RNA processing | 20/20 | 1/1 | 11/11 | 1/10 | 1/1 | 2/2 | **crov368** | DNA-directed RNA polymerase subunit alpha |
| **NCVOG0276** | **-** | Nucleotide metabolism | 13/20 | 1/1 | 2/11 | 10/10 | 1/1 | 2/2 | **crov452** | Ribonucleotide reductase small subunit |
| **NCVOG0278** | **-** | DNA replication, recombination and repair | 20/20 | 0/1 | 4/11 | 9/10 | 1/1 | 2/2 | **crov163** | RuvC, Holliday junction resolvase (HJRs); cl00243. Extended Pox_A22, Poxvirus A22 family (pfam04848). |
| **NCVOG0319** | **-** | Nucleotide metabolism | 15/20 | 1/1 | 0/11 | 1/10 | 1/1 | 2/2 | **crov161** | Thymidine kinase |
| **NCVOG0330** | **-** | Signal transduction regulation | 16/20 | 0/1 | 3/11 | 4/10 | 1/1 | 2/2 | **crov072** | RING-finger-containing E3 ubiquitin ligase (COG5432: RAD18) |
| **NCVOG1060** | **-** | DNA replication, recombination and repair | 20/20 | 0/1 | 11/11 | 1/10 | 1/1 | 2/2 | **crov037** | FLAP-like endonuclease XPG |
| **NCVOG1117** | **-** | Transcription and RNA processing | 20/20 | 1/1 | 1/11 | 8/10 | 1/1 | 2/2 | **crov212** | mRNA capping enzyme large subunit |
| **NCVOG1127** | **-** | Transcription and RNA processing | 0/20 | 1/1 | 0/11 | 7/10 | 1/1 | 2/2 | **crov230** | Transcription initiation factor IIB |

| **NCVOG id.** | **NCVOGs including proteins from all 45 analyzed viruses** | **Functional category** | **Number of genomes present in a cluster** | | | | | | | **NCVOG annotation** |
| --- | --- | --- | --- | --- | --- | --- | --- | --- | --- | --- |
|  |  |  | *Poxviridae* | *Asfarviridae* | *Irido- and Ascoviridae* | *Phycodnaviridae* | Marseillevirus | *Mimiviridae* | CroV |  |
| **NCVOG1164** | **-** | Transcription and RNA processing | 20/20 | 1/1 | 10/11 | 10/10 | 1/1 | 2/2 | **crov164** | A1L transcription factor/late transcription factor VLTF-2; pfam03295: Pox_TAA1; Poxvirus trans-activator protein A1 C-terminal |
| **NCVOG1353** | **-** | Nucleotide metabolism | 3/20 | 1/1 | 7/11 | 10/10 | 1/1 | 2/2 | **crov454** | Ribonucleoside diphosphate reductase, alpha subunit |
| **NCVOG0035** | **-** | DNA replication, recombination and repair | 3/20 | 0/1 | 2/11 | 0/10 | 0/1 | 2/2 | **crov462** | NAD+ dependent DNA ligase (smart00532)a |
| **NCVOG0059** | **-** | Other metabolic functions | 0/20 | 1/1 | 0/11 | 0/10 | 0/1 | 2/2 | **crov442** | FtsJ-like methyltransferase family proteins (pfam01728) |
| **NCVOG0246** | **-** | Other metabolic functions | 0/20 | 1/1 | 0/11 | 1/10 | 0/1 | 2/2 | **crov184** | pfam02902, Ulp1 protease family, C-terminal catalytic domain |
| **NCVOG0256** | **-** | Other metabolic functions | 20/20 | 0/1 | 0/11 | 0/10 | 0/1 | 2/2 | **crov138** | IMV envelope protein p35 |
| **NCVOG0267** | **-** | DNA replication, recombination and repair | 20/20 | 1/1 | 0/11 | 0/10 | 0/1 | 2/2 | **crov118** | RNA-helicase DExH-NPH-II |
| **NCVOG0329** | **-** | Other metabolic functions | 0/20 | 1/1 | 0/11 | 0/10 | 0/1 | 2/2 | **crov058** | UBCc, Ubiquitin-conjugating enzyme E2 (cd00195) |
| **NCVOG1192** | **-** | DNA replication, recombination and repair | 1/20 | 1/1 | 0/11 | 9/10 | 0/1 | 2/2 | **crov206** | YqaJ viral recombinase family (pfam09588) |
|  |  |  |  |  |  |  |  |  |  |  |
| **NCVOG1088** | **-** | Transcription and RNA processing | 0/20 | 1/1 | 11/11 | 0/10 | 1/1 | 0/2 | **crov480** | **RNA ligase (conserved in irido-, asfa- asco- and Marseille viruses)** |
| **NCVOG1068** | **-** | Nucleotide metabolism | 17/20 | 1/1 | 4/11 | 8/10 | 0/1 | 0/2 | **crov069** | **dUTPase (cl00493)** |
|  |  |  |  |  |  |  |  |  |  |  |
| **NCVOG0024** | **-** | DNA replication, recombination and repair | 0/20 | 1/1 | 0/11 | 0/10 | 1/1 | 2/2 | **0** | **Superfamily II helicase related to herpesvirus replicative helicase (origin-binding protein UL9), pfam03121** |
| **NCVOG1115** | **-** | Other metabolic functions | 20/20 | 0/1 | 0/11 | 0/10 | 1/1 | 2/2 | **0** | **Uracil-DNA glycosylase** |
| **NCVOG1361** | **-** | Uncharacterized | 2/20 | 1/1 | 4/11 | 1/10 | 1/1 | 2/2 | **0** | **pfam10544, T5orf172 domain** |
| **NCVOG0010** | **-** | Uncharacterized | 2/20 | 0/1 | 6/11 | 1/10 | 0/1 | 2/2 | **0** | **pfam02498: Bro-N; BRO family, N-terminal domain: This family includes the N-terminus of baculovirus BRO and ALI motif proteins.** |
| **NCVOG0036** | **-** | DNA replication, recombination and repair | 20/20 | 0/1 | 0/11 | 1/10 | 0/1 | 2/2 | **0** | **DNA topoisomerase I** |
| **NCVOG0040** | **-** | Other metabolic functions | 19/20 | 0/1 | 3/11 | 6/10 | 0/1 | 2/2 | **0** | **cd00127, DSPc, Dual specificity phosphatases (DSP); Ser/Thr and Tyr protein phosphatases** |
| **NCVOG0211** | **-** | Virion structure and morphogenesis | 20/20 | 1/1 | 11/11 | 0/10 | 0/1 | 2/2 | **0** | **Myristylated IMV envelope protein (pfam02442: Lipid membrane protein of large eukaryotic DNA viruses)** |
| **NCVOG1122** | **-** | Virion structure and morphogenesis | 20/20 | 0/1 | 9/11 | 0/10 | 0/1 | 2/2 | **0** | **Myristylated protein; pfam03003, DUF230** |
| **NCVOG1360** | **-** | Miscellaneous | 15/20 | 0/1 | 1/11 | 0/10 | 0/1 | 2/2 | **0** | **KilA domain (pfam04383); is always present at N-terminal except for mimiviruses. Sometimes is followed by a RING-finger domain** |
| **NCVOG1424** | **-** | Uncharacterized | 3/20 | 0/1 | 1/11 | 0/10 | 0/1 | 2/2 | **0** | **uncharacterized domain; found downstream KilA, BRO, and MSV199 domains. Also is found in some baculoviruses (gi 165969059, 18138388)** |
|  |  |  |  |  |  |  |  |  |  |  |
| **NCVOG0034** | **-** | DNA replication, recombination and repair | 11/20 | 1/1 | 0/11 | 6/10 | 1/1 | 0/2 | **0** | ATP-dependent DNA ligase (pfam01068, PRK01109)a |
| **NCVOG0009** | **-** | Host-virus interactions | 2/20 | 1/1 | 1/11 | 0/10 | 0/1 | 0/2 | **0** | pfam00653: BIR (Baculovirus Inhibitor of apoptosis protein Repeat) domain |
| **NCVOG0012** | **-** | Host-virus interactions | 18/20 | 1/1 | 0/11 | 1/10 | 0/1 | 0/2 | **0** | C-type lectin: smart00034, cd03594,cd03593, pfam00059, cd00037, pfam05966 |
| **NCVOG0320** | **-** | Nucleotide metabolism | 4/20 | 1/1 | 11/11 | 5/10 | 0/1 | 0/2 | **0** | pfam02223: Thymidylate kinase |

This table is based on data from supplementary tables of reference [6], and of reference [19]
