# Supplementary material for: The Giant Cafeteria roenbergensis Virus That Infects a Widespread Marine Phagocytic Protist Is a New Member of the Fourth Domain of Life
Source: PLoS One. 2011 Apr 29;6(4):e18935. doi: 10.1371/journal.pone.0018935 (PMC3084725; doi:10.1371/journal.pone.0018935)
Supplement: Table S4 — Presence/absence for COGs at least present in one NCLDV genome and that enable to distinguish between CroV and other NCLDVs. Footnote: +, presence; −, absence; COGs functional categories are those defined in the COG database [42]. (DOCX) [file pone.0018935.s016.docx]

**Table S4.** Presence/absence for COGs at least present in one NCLDV genome and that enable to distinguish between CroV and other NCLDVs.

| **COGs id.** | **Presence/absence of COGs in** | | | **COGs functional category** | **Functional annotation** |
| --- | --- | --- | --- | --- | --- |
|  | **CroV** | **Mimivirus** | **Marseillevirus** |  |  |
| COG0209 | **+** | **+** | + | F | Ribonucleotide reductase, alpha subunit |
| COG0207 | **+** | **+** | + | F | Thymidylate synthase |
| COG1435 | **+** | **+** | + | F | Thymidine kinase |
| COG0208 | **+** | **+** | + | F | Ribonucleotide reductase, beta subunit |
| COG0023 | **+** | **+** | + | J | Translation initiation factor 1 (eIF-1/SUI1) and related proteins |
| COG5053 | **+** | **+** | - | J | Translation initiation factor 4E (eIF-4E) |
| COG1405 | **+** | **+** | + | K | Transcription initiation factor TFIIIB, Brf1 subunit/Transcription initiation factor TFIIB |
| COG0571 | **+** | **+** | + | K | dsRNA-specific ribonuclease |
| COG0085 | **+** | **+** | + | K | DNA-directed RNA polymerase, beta subunit/140 kD subunit |
| COG0086 | **+** | **+** | + | K | DNA-directed RNA polymerase, beta' subunit/160 kD subunit |
| COG1594 | **+** | **+** | + | K | DNA-directed RNA polymerase, subunit M/Transcription elongation factor TFIIS |
| COG1758 | **+** | **+** | - | K | DNA-directed RNA polymerase, subunit K/omega |
| COG1061 | **+** | **+** | + | KL | DNA or RNA helicases of superfamily II |
| COG0553 | **+** | **+** | + | KL | Superfamily II DNA/RNA helicases, SNF2 family |
| COG0187 | **+** | **+** | + | L | Type IIA topoisomerase (DNA gyrase/topo II, topoisomerase IV), B subunit |
| COG0328 | **+** | **+** | + | L | Ribonuclease HI |
| COG0592 | **+** | **+** | + | L | DNA polymerase sliding clamp subunit (PCNA homolog) |
| COG3145 | **+** | **+** | + | L | Alkylated DNA repair protein |
| COG0258 | **+** | **+** | + | L | 5'-3' exonuclease (including N-terminal domain of PolI) |
| COG0417 | **+** | **+** | + | L | DNA polymerase elongation subunit (family B) |
|  |  |  |  |  |  |
| COG0675 | **+** | **+** | - | L | Transposase and inactivated derivatives |
| COG0272 | **+** | **+** | - | L | NAD-dependent DNA ligase (contains BRCT domain type II) |
| COG0470 | **+** | **+** | - | L | ATPase involved in DNA replication |
| COG0210 | **+** | **+** | - | L | Superfamily I DNA and RNA helicases |
| COG0550 | **+** | **+** | - | L | Topoisomerase IA |
| COG0514 | **+** | **+** | - | L | Superfamily II DNA helicase |
| COG0249 | **+** | **+** | - | L | Mismatch repair ATPase (MutS family) |
| COG1643 | **+** | **+** | - | L | HrpA-like helicases |
| COG3569 | **+** | **+** | - | L | Topoisomerase IB |
| COG5049 | **+** | **+** | + | LDA | 5'-3' exonuclease |
| COG0513 | **+** | **+** | - | LKJ | Superfamily II DNA and RNA helicases |

| **COGs id.** | **Presence/absence of COGs in** | | | **COGs functional category** | **Functional annotation** |
| --- | --- | --- | --- | --- | --- |
|  | **CroV** | **Mimivirus** | **Marseillevirus** |  |  |
| COG2131 | **+** | **-** | - | F | **Deoxycytidylate deaminase** |
| COG0232 | **+** | **-** | - | F | **dGTP triphosphohydrolase** |
| COG0034 | **+** | **-** | - | F | **Glutamine phosphoribosylpyrophosphate amidotransferase** |
| COG0756 | **+** | **-** | - | F | **dUTPase** |
| COG5257 | **+** | **-** | - | J | **Translation initiation factor 2, gamma subunit (eIF-2gamma; GTPase)** |
| COG1859 | **+** | **-** | - | J | **RNA:NAD 2'-phosphotransferase** |
| COG1601 | **+** | **-** | - | J | **Translation initiation factor 2, beta subunit (eIF-2beta)/eIF-5 N-terminal domain** |
| COG0532 | **+** | **-** | - | J | **Translation initiation factor 2 (IF-2; GTPase)** |
| COG0130 | **+** | **-** | - | J | **Pseudouridine synthase** |
| COG0060 | **+** | **-** | - | J | **Isoleucyl-tRNA synthetase** |
| COG1093 | **+** | **-** | - | J | **Translation initiation factor 2, alpha subunit (eIF-2alpha)** |
| COG5269 | **+** | **-** | - | JO | **Ribosome-associated chaperone zuotin** |
| COG2012 | **+** | **-** | - | K | **DNA-directed RNA polymerase, subunit H, RpoH/RPB5** |
| COG1737 | **+** | **-** | - | K | **Transcriptional regulators** |
| COG1224 | **+** | **-** | - | K | **DNA helicase TIP49, TBP-interacting protein** |
| COG0557 | **+** | **-** | - | K | **Exoribonuclease R** |
| COG0202 | **+** | **-** | - | K | **DNA-directed RNA polymerase, alpha subunit/40 kD subunit** |
| COG1243 | **+** | **-** | - | KB | **Histone acetyltransferase** |
| COG0454 | **+** | **-** | - | KR | **Histone acetyltransferase HPA2 and related acetyltransferases** |
| COG0338 | **+** | **-** | - | L | **Site-specific DNA methylase** |
| COG4581 | **+** | **-** | - | L | **Superfamily II RNA helicase** |
| COG2827 | **+** | **-** | - | L | **Predicted endonuclease containing a URI domain** |
| COG2452 | **+** | **-** | - | L | **Predicted site-specific integrase-resolvase** |
| COG2255 | **+** | **-** | - | L | **Holliday junction resolvasome, helicase subunit** |
| COG1796 | **+** | **-** | - | L | **DNA polymerase IV (family X)** |
| COG1570 | **+** | **-** | - | L | **Exonuclease VII, large subunit** |
| COG1111 | **+** | **-** | - | L | **ERCC4-like helicases** |
| COG0827 | **+** | **-** | - | L | **Adenine-specific DNA methylase** |
| COG0708 | **+** | **-** | - | L | **Exonuclease III** |
| COG0468 | **+** | **-** | - | L | **RecA/RadA recombinase** |
| COG0415 | **+** | **-** | - | L | **Deoxyribodipyrimidine photolyase** |
| COG0266 | **+** | **-** | - | L | **Formamidopyrimidine-DNA glycosylase** |
| COG0164 | **+** | **-** | - | L | **Ribonuclease HII** |

| **COGs id.** | **Presence/absence of COGs in** | | | **COGs functional category** | **Functional annotation** |
| --- | --- | --- | --- | --- | --- |
|  | **CroV** | **Mimivirus** | **Marseillevirus** |  |  |
| COG1208 | **+** | **-** | - | MJ | **Nucleoside-diphosphate-sugar pyrophosphorylase involved in lipopolysaccharide biosynthesis/translation initiation factor 2B, gamma/epsilon subunits (eIF-2Bgamma/eIF-2Bepsilon)** |
| COG0515 | **+** | **-** | - | RTKL | **Serine/threonine protein kinase** |
|  |  |  |  |  |  |
| COG0105 | **-** | **+** | - | F | **Nucleoside diphosphate kinase** |
| COG1503 | **-** | **+** | + | J | **Peptide chain release factor 1 (eRF1)** |
| COG0215 | **-** | **+** | - | J | **Cysteinyl-tRNA synthetase** |
| COG0162 | **-** | **+** | - | J | **Tyrosyl-tRNA synthetase** |
| COG0143 | **-** | **+** | - | J | **Methionyl-tRNA synthetase** |
| COG0018 | **-** | **+** | - | J | **Arginyl-tRNA synthetase** |
| COG1761 | **-** | **+** | - | K | **DNA-directed RNA polymerase, subunit L** |
| COG0419 | **-** | **+** | + | L | **ATPase involved in DNA repair** |
| COG0507 | **-** | **+** | + | L | **ATP-dependent exoDNAse (exonuclease V), alpha subunit - helicase superfamily I member** |
| COG0648 | **-** | **+** | + | L | **Endonuclease IV** |
| COG4294 | **-** | **+** | - | L | **UV damage repair endonuclease** |
|  |  |  |  |  |  |
| COG1428 | **-** | **-** | - | F | Deoxynucleoside kinases |
| COG1351 | **-** | **-** | - | F | Predicted alternative thymidylate synthase |
| COG0194 | **-** | **-** | - | F | Guanylate kinase |
| COG0216 | **-** | **-** | - | J | Protein chain release factor A |
| COG1793 | **-** | **-** | + | L | ATP-dependent DNA ligase |
| COG0270 | **-** | **-** | - | L | Site-specific DNA methylase |

+, presence; -, absence; COGs functional categories are those defined in the COG database [42]
